# NHS Health Check programme: a protocol for a realist review

**DOI:** 10.1136/bmjopen-2021-048937

**Published:** 2021-04-14

**Authors:** Claire Duddy, Geoff Wong, E W Gadsby, Janet Krska, Vivienne Hibberd

**Affiliations:** 1 Nuffield Department of Primary Care Health Sciences, Oxford University, Oxford, UK; 2 Centre for Health Services Studies, University of Kent, Canterbury, UK; 3 Universities of Greenwich and Kent, Medway School of Pharmacy, Chatham Maritime, UK; 4 Public Involvement in Pharmacy Studies Group, University of Kent Medway School of Pharmacy, Chatham Maritime, UK

**Keywords:** risk management, preventive medicine, public health

## Abstract

**Introduction:**

The NHS Health Check aims to identify individuals at increased risk of cardiovascular diseases (CVDs) among the adult population in England. The Health Check includes calculation of CVD risk and discussion of pharmacological and lifestyle approaches to manage risk, including referral to lifestyle support services. The programme is commissioned by Local Authorities (LAs) and is delivered by a range of different providers in different settings. There is significant variation in activity, with uptake ranging from 25% to 85% in different areas, and clear evidence of variation in implementation and delivery practice.

**Methods and analysis:**

We aim to understand how the NHS Health Check programme works in different settings, for different groups, so that we can recommend improvements to maximise intended outcomes. To do so, we will undertake a realist review and a survey of LA public health teams. Our review will follow Pawson’s five iterative stages: (1) locate existing theories, (2) search for evidence, (3) article selection, (4) extract and organise data and (5) synthesise evidence and draw conclusions. Our review will include documents describing local implementation alongside published research studies. We will recruit a stakeholder group (including Public Health England, commissioners and providers of Health Checks, plus members of the public and patients) to advise us throughout. Our survey will be sent to all 152 LAs in England to gather detailed information on programme delivery (including COVID-19-related changes) and available referral services. This will enable us to map delivery across England and relate these data to programme outcomes.

**Ethics and dissemination:**

Ethical approval is not required for this review. For the survey, we have received approval from the University of Kent Research Ethics Committee. Our findings will be used to develop recommendations on tailoring, implementation and design strategies to improve delivery of the NHS Health Check in different settings, for different groups.

**PROSPERO registration number:**

CRD42020163822.

**Strengths and limitations of this study**

- This is the first realist review of the NHS Health Check and will improve our understanding of how the programme works in different settings and for different groups.
- In addition to including published research studies on the Health Check programme, this review will draw on learning from documents that describe local implementation and innovation in programme delivery.
- Our review will be augmented by a comprehensive survey of local public health teams, capturing new data on current delivery models, including recent innovation in response to the COVID-19 pandemic.
- Our review may be limited by the richness and relevance of evidence available in the literature.
- Survey response rates may be adversely affected by COVID-19 pressures, and we will need to take steps to mitigate these wherever possible.

## Background

Cardiovascular disease (CVD) causes a quarter of all deaths in the UK and is the largest cause of premature mortality in deprived areas. Early detection and prevention is an important priority for the National Health Service (NHS) in England, and the NHS Long Term Plan (2019) commits to taking wider action on prevention to tackle the underlying causal factors. Over the course of 2020, it has also become clear that many risk factors associated with CVD, and existing health inequalities, are associated with poorer outcomes for patients with COVID-19.^1^ The UK government has therefore highlighted the potential role that the NHS Health Check may have to play in helping to address these risk factors.^2^

The NHS Health Check programme is one of the main pillars of CVD prevention efforts in England. It was first launched in 2009, aiming to offer a 5-yearly assessment of individual risk of developing coronary heart disease, stroke, diabetes and chronic renal disease to the population aged between 40 and 74 years.^3^ The Health Check involves measurement of key risk factors and calculation of CVD risk, followed by discussion and agreement on lifestyle and pharmacological approaches to managing the risk. This is a national, mandated programme, originally commissioned by Primary Care Trusts across England. The NHS Health Check programme was relaunched in 2013, when commissioning moved from Primary Care Trusts to Local Authorities (LAs), with an implementation review and action plan.^4^ Although tests and measurements are standardised to help ensure the safety, quality and effectiveness of the programme,^5 6^ LAs have flexibility in how and who they commission to provide NHS Health Checks. As commissioning and delivery are determined locally, with the aim of meeting the needs of local populations, there is inevitable variation in delivery, uptake and outcomes.

The total eligible population for the Health Check programme has been estimated to be about 15.5 million.^7^ The largest and most recently published analysis of national data relating to the programme found that almost 10 million eligible people were offered an NHS Health Check between 2012 and 2017.^8^ Of these, 52.6% (just over 5 million) took up the offer. Although national uptake rates generally increased over this period, there was significant regional variation, with uptake rates calculated for upper-tier LA areas ranging from 25.1% to 84.7%.^8^ These findings are in line with previous analyses that have identified significant variation in invitation and uptake rate for the programme,^9^ and variation in Health Check delivery and follow-up, including referrals to lifestyle services.^10 11^ At present, minimal national data exist to explain these differences, yet it is important to determine what does work, for whom and in what setting.

At the time of writing, Public Health England (PHE) is undertaking a review of the NHS Health Check, with the aim of making recommendations to improve both the content and delivery of the programme.^12 13^ The review will encompass a wide remit and include consideration of whether additional ‘checks’ should be incorporated into the current offering, options to tailor the Health Check and personalise the programme for individuals, and digital tools that may offer opportunities to improve delivery. This review is being undertaken in a wider context of considerable uncertainty for national-level public health functions, following the announcement by Government in August 2020 that PHE will be dismantled and a new national institute for health protection created.^14 15^

### Overview of existing evidence

In 2014, PHE established an Expert Scientific and Clinical Advisory Panel (ESCAP) to continually review the evidence on the NHS Health Check programme.^16^ This group recommended periodic syntheses of published evidence. The first such review was a rapid evidence synthesis, published in 2017^11^ and updated in 2020.^10^ These reviews addressed six research questions identified by PHE, focused on uptake (questions 1–3), management of those at high risk (question 4), patient experience (question 5) and a specific set of outcomes related to the Health Check, including disease detection, referral, reductions in CVD risk and prescribing (question 6). The authors of both reviews identified significant gaps in the literature, and both ESCAP^17^ and the review authors made recommendations for action and further research. These recommendations included the following:

1. Improved characterisation of local variations in implementation of the Health Check, to allow comparisons and sharing of best practice.^11^
2. The need to develop a model that fully reflects the real-life NHS Health Check intervention and draws on current evidence to estimate its impact.^10 17^
3. The need for more research to determine the effect of the Health Check on lifestyle behaviour in different groups.^17^

These rapid evidence syntheses included documents identified by PHE using a systematic and comprehensive search strategy, updated each quarter.^18^ Searching was conducted in multiple databases: MEDLINE, PubMed, Embase, HMIC, CINAHL, Global Health, PsycINFO, the Cochrane Library, NHS Evidence, Google Scholar, Google, Clinicaltrials.gov and the ISRCTN registry. The rapid reviews augmented these with additional searches in the Web of Science (Science Citation Index),^10 11^ OpenGrey^11^ and a review of abstracts submitted to the 2017 PHE NHS Health Check conference. Together, the two reviews include evidence covering the period January 1996 to December 2019.

In addition to these reviews (and their associated academic publications^9 19–21^), a further three reviews relating to the NHS Health Check have been published to date,^22–24^ and we have also identified two other systematic reviews focused on participation and patient experience in similar prevention programmes.^25 26^ Drawing on the existing evidence, below we summarise what is currently known about the NHS Health Check programme:

- **Coverage (proportion eligible who receive a Health Check**) is known to vary substantially across regions and settings, but is consistently higher in older people, women and in more deprived populations, although this may reflect targeting.^11^ Studies suggest that community outreach services can reach particular sociodemographic groups,^27^ but one study suggests that these services may create inaccuracies in reporting.^28^
- **Uptake (attendance following invitation**) varies across regions and at general practitioner (GP) practice level.^24^ The evidence on uptake in different groups is highly heterogeneous. There is relatively consistent evidence that older people and women are more likely to take up invitations, but mixed findings in relation to ethnicity and deprivation, with some studies showing higher uptake in specific groups, whereas others show no difference.^10 11 24^ There is also clear evidence that uptake is lower among smokers.^11 24^
- **Invitations** are issued in different formats, though letters are the most common.^29 30^ Recent studies of the effectiveness of different formats have found that modifications to standard letters, text message invitations/reminders, telephone and opportunistic face-to-face invitations can increase uptake.^10 11 29 31^ One cross-sectional study suggests that different invitation methods may be more or less effective for different ethnic and gender groups.^29^ Telephone calls including the option to book an appointment during the call may overcome anticipated difficulties in making appointments and offer an opportunity to increase participants’ understanding of the Health Check,^32^ which may be barriers to uptake.^23^ Other barriers may include aversion to preventive medicine, competing priorities and, for community pharmacy and outreach settings, concerns about privacy and confidentiality.^10 11 19 23^ Some qualitative evidence highlights the convenience of these settings and the value of community ambassadors.^10 20^
- **Delivery** of the Health Check varies considerably across settings, despite the standardisation provided by PHE’s guidance and the legislation that mandates its delivery. Providers delivering NHS Health Checks have reported challenges with workload, nformation technnology (IT), funding, training and the need to cover multiple aspects within one consultation.^10 11 23 33^ A recent review found that although many providers recognised the importance of behaviour change to reduce CVD risk, professionals have different views on the contributions of behavioural versus pharmacological interventions and on the clinical and cost-effectiveness of the Health Check programme.^23^ Providers recognise the difficulty patients face in making sustained behaviour and lifestyle changes, acknowledging the need to take patients’ social circumstances and resources into account.^34 35^ Professionals have also expressed concerns about limited access to appropriate lifestyle services for onward referrals and about stretched resources and workload in primary care.^10 11^
- **Patient experiences** are reported to be positive overall, with patient surveys indicating high levels of ‘patient satisfaction’ (consistently over 80%) and some reporting that attendance had precipitated lifestyle changes.^10 11^ However, qualitative studies have found that some patients report unmet expectations and confusion around follow-up and risk scores. Some patients have found lifestyle advice too simplistic and unpersonalised.^10 11^
- **Outcomes** demonstrating clinical and/or cost-effectiveness of the NHS Health Check are harder to obtain. Existing research demonstrates that the Health Check increases the detection of CVD risk factors and disease, and leads to increased statin prescribing (by 3%–4%).^10 11^ Some studies also report increased prescribing of antihypertensive drugs (but one cohort study reported that Health Check attendees were *less* likely to receive antihypertensives than matched controls^36^). Three national studies found that the Health Check increased referrals to smoking cessation, weight management, exercise or alcohol support services.^36 37^ However, regional studies demonstrate wide variation in service availability and referral practice across England.^10 11^ The PHE-commissioned rapid reviews identified six primary studies that examined behaviour change, but smoking is the only health behaviour assessed. A limited number of studies have demonstrated post-Health Check improvements in relevant risk factors, including body mass index, diastolic blood pressure, total cholesterol and overall CVD risk, but results across studies are inconsistent and some have found no evidence of any effect.^10^

It is clear across the existing reviews that the emphasis in the literature is on the early steps of the Health Check pathway, and especially on the invitation to and uptake of the Health Check. This focus may reflect the variation that is apparent in the published indicators on Health Checks^7^ and the emphasis on improving uptake in the legislation that mandates the programme.^38^ Studies of Health Check delivery focus on patient and provider experience and perceptions, and there is more limited evidence on what happens after a Health Check. There is a notable absence of studies of post-Health Check behaviour change beyond smoking cessation. At all stages, the existing research demonstrates wide variation in implementation and practice, and significant uncertainties in relation to understanding this variation and the optimal strategies for increasing coverage and uptake, delivery models and maximising important patient outcomes.

### Evidence explaining why this research is needed now

Since publication of the rapid evidence review in February 2017, and the rapid review update in 2020, several new studies have been published, which add to the findings of the review. A PubMed search conducted in November 2020 for studies published since December 2019 has identified a further eight empirical studies concerning the NHS Health Check^8 39–45^ and one protocol for an implementation study and trial.^46^

To improve our understanding of how the NHS Health Check achieves its outcomes, it is essential to learn as much as possible from how the programme is delivered, in different settings and by different providers. In the past, case studies have been used by PHE to illustrate ‘good practice’.^4^ To date, case studies shared by PHE on the Health Checks website (n=24 in November 2020) have focused on sharing practice in relation to increasing coverage or targeting Health Check invitations to particular groups.^47^ Selected local evaluations submitted to PHE have also been shared on the website (n=26 in November 2020),^48^ and learning from local implementation of the Health Check programme has regularly been shared at PHE-run conferences focused on the Health Check and CVD prevention.^49^ In addition, a survey of commissioners and providers focused on targeting Health Checks was recently carried out by PHE,^50^ and a further survey seeking data on local delivery models is currently underway. Both add further local learning which could be used to help understand what works, for whom, how and in what setting.

None of the reviews conducted to date have effectively used this abundant learning from the local level. The first rapid review looked at abstracts submitted to the 2017 NHS Health Check conference, but these form a minor aspect of the review.^11^ More recently, PHE commissioned the University College London Centre for Behaviour Change to conduct a review of barriers and facilitators to behaviours relevant to NHS Health Checks, including those of providers and invitees/attenders; again, this review included only papers published in academic journals.^23^

It is now 11 years since the Health Check programme was launched. The amassed evidence from small, local unpublished studies needs to be combined with the published papers, many of which also cover only one locality. This evidence also needs to be combined with more comprehensive knowledge of the variety of ways in which different localities implement the NHS Health Check programme. It is by combining and analysing this evidence that we seek to answer the important research questions set out below.

### Aim and objectives

The NHS Health Check programme is arguably one of the largest prevention programmes of its type in the world and a cornerstone for the NHS prevention programme. However, many unanswered questions remain. To ensure that our research is sufficiently focused and will produce findings that are relevant to knowledge users, we sought advice and feedback from colleagues at PHE and our wider stakeholder group to arrive at our aims, objectives and research questions.

#### Aim

The aim of this study was to understand how the NHS Health Check programme works in different settings, for different groups, to recommend improvements to maximise intended outcomes.

#### Objectives

1. To conduct a realist review to enable understanding of how the NHS Health Check programme works in different settings, for different groups, to achieve its outcomes.
2. To map how the programme is currently delivered across England, using data collected in a PHE survey (in October 2020) and data we will collect using our own online survey of LAs.
3. To provide recommendations on tailoring, implementation and design strategies to improve the current delivery and outcomes of the NHS Health Check programme in different settings, for different groups.

#### Review questions

1. What are the mechanisms by which the current NHS Health Check programme produces its intended outcomes?
2. What are the important contexts which determine whether the different mechanisms produce intended outcomes?
3. In what circumstances are such interventions likely to be effective?

## Methods and analysis

### Objective 1: to conduct a realist review

**Figure 1:**
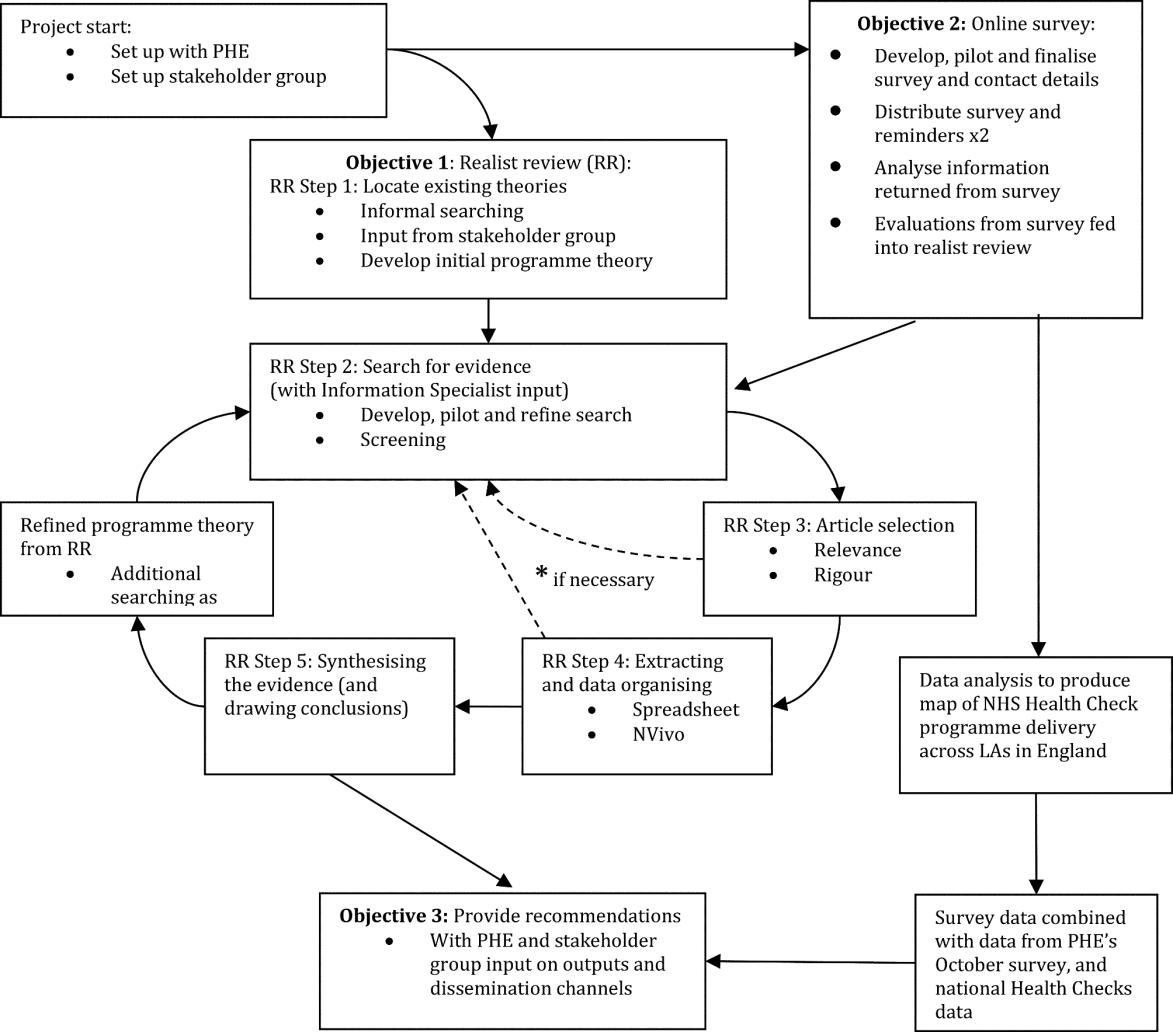
Project flow diagram. LAs, Local Authorities; NHS, National Heath Service; PHE, Public Health England.

The plan of investigation will follow this protocol which is informed by Pawson’s five iterative stages in realist reviews (see figure 1).^51^ We have chosen to use a realist review approach because the existing research indicates that the NHS Health Check programme is a complex intervention that has a range of outcomes (eg, variable rates of attendance, follow-up, onward referral, prescription) which are context-sensitive and vary for different groups. We are also aware that the NHS Health Check programme is continually evolving: work exploring the potential for digital services has been under consideration since 2017,^52^ and a wide-ranging review of the Health Check programme is currently underway and expected to make recommendations in early 2021.^12^ In addition to this, it is clear that the pause and restart of the Health Check programme during the COVID-19 pandemic have provoked a range of responses at local levels, including the introduction of new delivery models in some areas.^53^

Any evidence synthesis that seeks to make sense of how to improve the tailoring, implementation and design of the NHS Health Check programme must take into account the various contexts and interventional strategies in which NHS Health Checks are delivered and the varied outcomes for different groups. A realist review will be able to generate the knowledge needed to address both these issues.^54^ Realist review is an interpretive, theory-driven approach to synthesising evidence from qualitative, quantitative and mixed-methods research. Its main strength comes from providing findings that coherently and transferably explain how and why context can influence outcomes.

**Figure 2:**
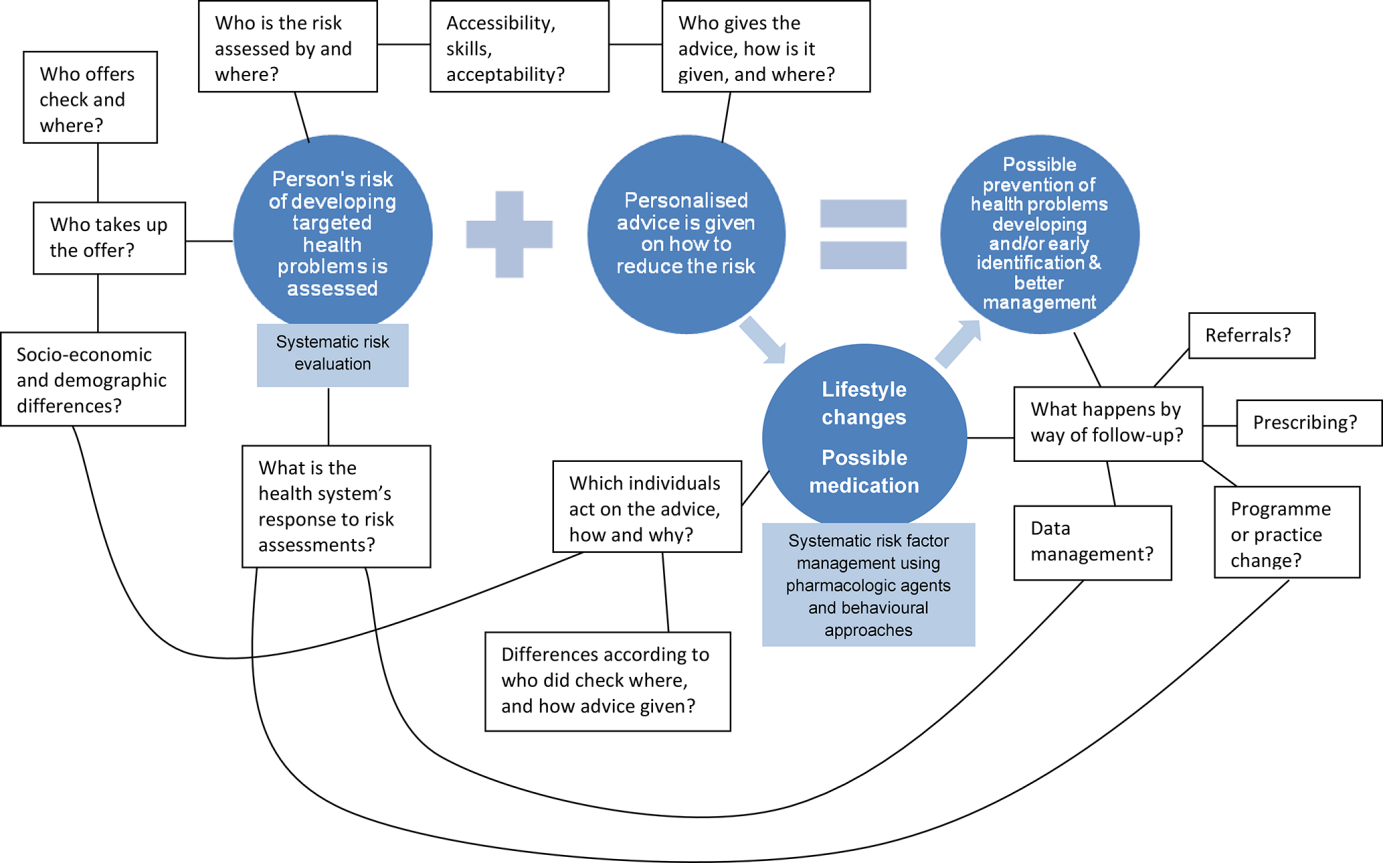
Initial programme theory behind NHS Health Checks and some of the processes that may influence outcomes. NHS, National Health Service.

This process of explanation building starts with the development and refinement of a realist ‘programme theory’ of the NHS Health Check programme. To do this, we have ‘mapped’ the sequence of steps needed to achieve the final intended outcomes for the programme, taking account of the processes outlined by PHE in their implementation guidance (see figure 2). This initial programme theory will be refined (see step 1 below) and then further refined and tested against empirical evidence during the review (see steps 2–5).

### Patient and public involvement

Throughout the review, we will consult with a wide range of stakeholders with content expertise and a variety of perspectives. We will invite policy-makers, commissioners, training providers and front-line providers of the Health Check programme, as well as representatives from relevant charities and members of the public drawn from the eligible population. We will update and extend the membership of this group as needed over the course of the review. We have secured the participation of PHE as a key stakeholder, and it has expressed strong interest in assisting us during the review and in our findings. Our initial programme theory will be presented to our stakeholder group and refined based on their feedback. As the programme theory is further refined over the course of the review, the stakeholders will be regularly consulted on the developing findings. The group will meet (either virtually or face to face) four times during the study and communicate via email as necessary. The meetings will be chaired by GW or VH (our PPI Lead, a member from the Public Involvement in Pharmacy Studies at Medway School of Pharmacy group).

This review protocol has been registered with PROSPERO and will follow current quality and publication standards.^55^ An overview of the project may be found in figure 1, and the main steps involved in the review are outlined in more detail below.

#### Step 1: locate existing theories

The goal of this step is to identify theories that explain how the NHS Health Check programme is supposed to work (and for whom), when it does work, when it does not achieve desired changes in clinical practice and patient outcomes, why it is not effective and why it is not being used.^56^ The rationale for this step is that interventions are ‘theories incarnate’—that is, underpinning the design of such interventions are theories of why certain components are required. In other words, the designers of the programme have put it together in a certain way based on their theories about what needs to be done to get one or more desired outcomes.^57^ For example, one theoretical assumption underpinning the programme design is that once patients are given information about their cardiovascular risk score, this knowledge will motivate a proportion of them to make the necessary lifestyle changes to reduce it.^58^ There are a number of different theories and models that support such an assumption, although evidence highlights the complexity of achieving sustained behaviour change.^59^

To locate relevant theories, we will iteratively

1. consult with key content experts in our stakeholder group, and
2. informally search the literature to identify existing theories, including both grey literature in the form of NHS Health Check programme documentation and published research that has employed formal or substantive theory to understand the programme.

This informal searching differs from the more formal searching process described below in step 2; it is exploratory and aims to quickly identify a range of possible explanatory theories that may be relevant. Search methods such as citation tracking and snowballing,^60^ along with more structured searching for theories,^61 62^ will be used.

From these, we will refine an initial programme theory to test in the review. A first version of this initial programme theory is outlined as a starting point in figure 2. In step 1, we will refine this model within the project team and present it to our stakeholder group for their feedback.

##### Focus of the review

To develop this protocol, we undertook searches to identify existing reviews related to the Health Check programme. In so doing, we identified an existing focus on the early steps in our initial programme theory, and especially on *invitation* and *uptake* of the NHS Health Check (see figure 2). Conversely, there has been less focus on what happens next, in relation to follow-up, onward referral and ongoing support for lifestyle and behaviour change. As a result, we have decided to focus our review efforts on these later steps, which are crucial in delivering the programme’s intended outcomes, in relation to supporting individuals to make changes to their behaviour and lifestyle, and ultimately to reduce their risk of experiencing a heart attack, stroke or developing some forms of dementia.^6^ We have confirmed the value of this focus with PHE, our key stakeholder, and we will seek further feedback and advice from our wider stakeholder group to help us to develop our thinking about this stage of the Health Check pathway. However, in recognition of the complex nature of the Health Check programme and the possibility that feedback loops may exist, such that the characteristics of one step may be inextricably bound up with others, we will not exclude evidence relating to any other programme steps, but will actively seek out data that might shed light on relationships between each step.

As noted above, we are also aware that the Health Check programme is continually evolving, and especially aware of the effects of the COVID-19 pandemic on programme delivery, and that a major review of the programme began in August 2020. PHE and the Department of Health and Social Care have acknowledged that many of the factors assessed by the NHS Health Check are also risk factors for severe COVID-19 and the fact that the programme has an explicit aim to reduce health inequalities,^2 13^ now a pressing agenda. In addition, restrictions on face-to-face contact and increased pressure on health services have created a new impetus to adapt programme delivery and may speed up the proposed shift to introduce digital methods or other innovations. Our own review project is taking place within this fast-moving context, and as such, we will aim to prioritise the inclusion of evidence that can support findings and recommendations with relevance for the ‘new normal’.

#### Step 2: search for evidence

##### Formal search

The purpose of this step is to find a relevant ‘body of literature’ with which to further develop and refine the initial programme theory from step 1. The search strategy used will be designed, piloted and conducted by CD, in collaboration with the project team. CD is an information specialist with extensive experience of conducting searches for complex systematic reviews, particularly realist reviews.

At the outset of this project, we were aware that PHE regularly undertakes literature searching for new evidence relating to the NHS Health Check. These regular searches employ a very sensitive search strategy across 13 relevant sources (PubMed, MEDLINE, Embase, HMIC, CINAHL, Global Health, PsycINFO, Cochrane Library, NHS Evidence, Google Scholar, Google, Clinicaltrials.gov and the ISRCTN registry).^18^ These searches have been used in previous review projects commissioned by PHE to identify evidence relating to the NHS Health Check.^10 11^ These reviews have included additional searches in OpenGrey and/or Web of Science (Science Citation Index); the most recent review captured studies published until the end of December 2019.^10^

We do not intend to duplicate this work, but instead aim to reuse and extend it as follows:

1. We will reuse existing searches by considering for inclusion all documents included in the two existing reviews commissioned by PHE (n=98 documents). These were empirical (quantitative and qualitative) studies of the NHS Health Check. The eligibility criteria employed in each of these reviews are summarised in table 1.
2. We will then seek additional material to consider for inclusion:
  - We will run additional targeted searches focused on identifying material related to the NHS Health Check that was excluded from the existing reviews. This may include, for example, relevant commentary or opinion, which are not excluded from realist reviews as they may contribute to theory building; studies focused on aspects of the Health Check not covered by the existing reviews; and studies published since December 2019. We will use specific searches to identify additional documents focused on the Health Check programme in England, using specific free-text terms describing the Health Check programme alongside relevant subject heading terms as appropriate. We will search MEDLINE, Embase, CINAHL, HMIC and Web of Science (Science and Social Science Citation Indexes). The full details of the search strategies for these searches are available in the online supplemental file.
  - We will trawl the NHS Health Checks website for documents including case studies (n=24 in November 2020), local evaluations (n=24 in November 2020) and abstracts and posters presented at the Health Checks/Cardiovascular Disease Prevention annual conferences from 2014 to 2020 (n>450), where these are accessible via current and archived versions of the website. These sources represent an important source of data on local implementations of the Health Check that has been excluded from previous reviews.
  - For each included published document, we will undertake backward and forward citation searches, that is, for these documents we will screen their reference lists, and use Google Scholar’s ‘cited by’ links to identify future documents that cited these. Where necessary, we will also undertake further searching to identify ‘sibling’ documents, related to those that we have already included by virtue of being part of the same broader research projects.^62^
3. If necessary (ie, if further data are required for programme theory development), we will continue to seek additional material as follows:
  - We will consider for inclusion all additional studies focused on Health Check (and related international programmes and evidence) included in PHE’s published quarterly literature reviews from October 2014 onwards available on the Health Checks website (including those published during our own review project, to ensure the most up-to-date material was included).
  - We will run more targeted searches for additional grey literature, including, for example, searching additional relevant websites (Department of Health and Social Care, NHS England, Clinical Commissioning Groups and LAs).
  - We will seek access to unpublished evaluation reports by contacting LA public health teams directly (see objective 2 below for more details).

10.1136/bmjopen-2021-048937.supp1 Supplementary data

**Table 1:** Summary of eligibility criteria for commissioned rapid reviews of the NHS Health Check

Inclusion criteria	Exclusion criteria
Studies of the NHS Health Check Populations including those eligible for, attending, not attending and providing NHS Health Checks **Study designs:** RCTs, quasi-RCTs Before-and-after studies with appropriate comparator groups Interrupted time series Cohort studies (prospective or retrospective) Case–control studies Qualitative studies using recognised methods Economic and health outcome modelling	Editorials, commentaries and opinion pieces

NHS, National Health Service; RCT, randomised controlled trial.

To ensure we keep up to date with emerging material as the review progresses, we will set up a regular search alert (via Google Scholar) and continue to consult PHE’s regularly published literature reviews (via the Health Checks website).

##### Screening

For the material and searches described above, our inclusion and exclusion criteria are broad as we seek to find quantitative, qualitative and mixed-methods documents, and relevant grey literature. The following criteria will be applied:

###### Inclusion

- Intervention: NHS Health Check programme (all delivery models, including face to face and digital).
- Study design: all study designs.
- Setting: any setting providing NHS Health Checks in England.
- Participants: all adults eligible for NHS Health Checks.
- Outcome measures: all outcome measures related to NHS Health Checks.

###### Exclusion

- Cardiovascular screening programmes run in countries other than England.
- Other NHS screening programmes.
- Routine health checks offered to specific target populations by the NHS which are not part of the NHS Health Check programme.

Screening will be undertaken by CD, based on title and abstract, with a 10% random sample of the citations retrieved from searching being reviewed independently for quality control by GW. Where necessary (eg, no abstract is available), the full text of a document will be consulted. Any disagreements about inclusion will be resolved by discussion. If disagreements remain, the matter will be presented to JK and resolved by majority vote.

##### Additional searching

An important process in realist reviews is searching for additional data to inform programme theory development. In other words, more searches may be undertaken if we find that we require more data to develop and test certain parts of our programme theory. Some of our proposed strategies for identifying additional data are listed above (point 3), but this list is not necessarily exhaustive. As our programme theory will take into account the wider contextual factors that impact outcomes from the NHS Health Check, we may also run searches to identify additional data on relevant contexts. For example, we anticipate that we may need to seek evidence relating to the commissioning process, relationships between Health Check providers (including GPs) and LAs, interprofessional relationships, and the presence of other prevention programmes and related services, which may interact or overlap with the Health Check.

This additional searching will greatly increase the amount of relevant data available to us for the realist review. For any additional searching undertaken, the project team will discuss and set inclusion and exclusion criteria. CD will develop, pilot and refine additional search strategies as needed. Screening will be conducted as described above. As in step 1, these searches are likely to be exploratory and purposive, potentially seeking documents from a wide range of disciplines. Where applicable, we will follow search strategies described by Booth *et al*, developed for just such data.^62^

#### Step 3: article selection

Following initial screening, documents will be read in full text and selected for inclusion in the review based on an assessment of *relevance* (whether data can contribute to theory building and/or testing) and *rigour* (whether the methods used to generate the relevant data are credible and trustworthy).^63^ Even when a document found from the initial search has been screened and has met the inclusion criteria, it may still not contain any data that are relevant for programme theory development and refinement.

CD will read the full text of all the documents that have been included after initial screening. Documents will be selected for inclusion when they contain data that are relevant to the realist analysis—that is, could inform some aspect of the programme theory. At the point of inclusion based on relevance, an assessment will also be made of rigour (how trustworthy were methods used to generate the data). To illustrate how we will operationalise the assessment of rigour: if data have been generated using a questionnaire/survey, the trustworthiness of the data will be considered to be higher if the questionnaire had previously been shown to be reliable and valid, and remained unaltered (or where subsequent testing had been undertaken following any alterations). However, data may still be included even if judged to be of limited rigour, as we will also make an overall assessment of rigour at the level of the programme theory.^64^ In other words, we will also consider the role that each piece of data plays in our developing programme theory and how it strengthens (or not) our explanations of outcomes.

A random sample of 10% of documents identified as including relevant data will be selected, assessed and discussed between CD and GW to ensure that decisions to finally include have been made consistently. The remaining 90% of decisions will be made by CD (though a number of these may require further discussion and joint reading within the project team, where there is any uncertainty over issues of relevance or rigour). As necessary, we will employ the same decision-making processes as were used during screening in step 2.

#### Step 4: extracting and organising data

The main characteristics (bibliographic details and information relating to study design, participants, settings and findings) of the included documents will be extracted into an Excel spreadsheet.

The full text of included documents will be uploaded into NVivo (a qualitative data analysis software tool). Relevant sections of text in these documents will be coded in NVivo and interpreted as relating to contexts, mechanisms and outcomes, or relationships between these. Coding will be deductive (codes created in advance of data extraction and analysis will be informed by the initial programme theory), inductive (codes will be created to categorise data reported in included studies) and retroductive (codes will be created based on an interpretation of data, to infer the causal forces that generate observed outcomes, ie, mechanisms). Each new element of data will be used to refine the theory if appropriate, and as the theory is refined, included studies will be rescrutinised to search for data relevant to the revised theory that may have been missed.

Data extraction and organisation will be undertaken by CD. As with screening and inclusion decisions, a random sample of 10% of documents will be independently checked by GW for quality control. Any disagreements will be resolved by discussion, and if disagreements remain, JK will be asked for her opinion, and resolution will be by majority vote.

#### Step 5: synthesising the evidence and drawing conclusions

We will use a realist logic of analysis to make sense of data included in the review. CD will undertake this step with support from GW and EG. We will use a series of questions about the relevance and rigour of content within data sources as part of our process of analysis and synthesis^65^:

- Relevance: Are sections of text within this document relevant to programme theory development?
- Rigour (judgements about trustworthiness): Are these data sufficiently trustworthy to warrant making changes to any aspect of the programme theory?
- Interpretation of meaning: If the section of text is relevant and trustworthy enough, do its contents provide data that may be interpreted as functioning as context, mechanism or outcome?
- Interpretations and judgements about context–mechanism–outcome–configurations (CMOCs): What is the CMOC (partial or complete) for the data that have been interpreted as functioning as context, mechanism or outcome? Are there further data to inform the particular CMOCs contained within this document or other documents? If so, which other documents? How does this particular CMOC relate to other CMOCs that have already been developed?
- Interpretations and judgements about programme theory: How does this particular (full or partial) CMOC relate to the programme theory? Within this same document are there data which inform how the CMOC relates to the programme theory? If not, are there data in other documents? Which ones? In light of this particular CMOC and any supporting data, does the programme theory need to be changed?

Data to inform our interpretation of the relationships between contexts, mechanisms and outcomes will be sought not just within the same document, but across documents (eg, mechanisms inferred from one document could help explain the way contexts influenced outcomes in a different document). Synthesising data from different documents is often necessary to compile CMOCs, as not all parts of the configurations will always be articulated in the same document.

Within the analytic process set out above, we will use interpretive cross-case comparison to understand and explain how and why reported outcomes have occurred, for example, by comparing the elements within the NHS Health Check programme which have produced a particular outcome against those which have not, to understand how context has influenced reported findings. When working through the questions set out, where appropriate we will use the following forms of reasoning to make sense of the data:

- Juxtaposition of data: for example, where data about how Health Check setting influenced outcome in one document enable insights into data about outcomes in another document.
- Reconciling of data: where data differ in apparently similar circumstances, further investigation is appropriate to find explanations for why these differences have occurred.
- Adjudication of data: where there are conflicting data, plausibility of these data can be informed on the basis of methodological strengths or weaknesses of the data collection methods.
- Consolidation of data: where outcomes differ in particular contexts, an explanation can be constructed of how and why these outcomes occur differently.

In addition to the material identified for inclusion in the review, additional information obtained via a survey will also provide rich data on contexts and mechanisms in different localities, which will enhance our ability to make sense of the data from the documents identified in the searches. See objective 2 for more details.

### Objective 2: to map current delivery across England

This objective will both enable additional material (local knowledge, unpublished evaluations and examples of best practice and COVID-19-related innovation) to be identified for the review and provide a comprehensive overview of how different localities across England implement the NHS Health Check programme. It will be conducted (with support from the project team and stakeholder group) by EG, a senior public health researcher with a strong understanding of LA commissioning and public health service provision.

PHE conducted a survey of LA commissioners in October 2020 as part of the wider review of the Health Check programme. From this survey, they have provided detailed information from the 104 responding authorities related to some aspects of the programme’s delivery from April 2019 until March 2020, in particular: how the eligible population was identified; what methods were used for first invitation; when and in which settings Health Check appointments were made available and who provided them; whether and what type of point of care testing was used; whether digital solutions were used in delivery; and how the provider workforce was supported. PHE and its survey respondents have granted us permission to include these data in our study.

We will supplement this existing information with our own survey, by asking questions related to current delivery models (in 2021, following the COVID-19-related pause to the service) and questions related to options for onward referral and follow-up of patients after the Health Check encounter. In particular, our survey will identify the extent to which commissioners and providers are changing the way they commission and deliver the NHS Health Check programme in light of the COVID-19 pandemic. We will also identify the extent to which services are available to support those identified as having modifiable risk factors, which will help us to address our review focus on what happens after a Health Check, especially in relation to follow-up, onward referral and ongoing support for lifestyle and behaviour change.

Our survey will be designed in collaboration with our stakeholders and pilot tested prior to being distributed as an online survey (using Jisc Online Surveys) to all 152 upper-tier and unitary LAs in England. To ensure a maximal response and to ensure the survey is correctly targeted to those who can answer it, we will work closely with PHE to distribute the survey and make use of its tried-and-tested processes for dissemination. PHE has agreed to send the survey on our behalf through its local networks and send up to two reminders to non-responders. It will also publicise it through its established national and local communications channels (including the NHS Health Check e-Bulletin, website and Twitter feed).

Survey responses will be logged, managed and sorted for analysis using Microsoft Excel. Where there are missing data from completed surveys, we will search each LA’s website to see whether we can find the necessary information. Resources and time permitting, we will also search for data from the websites of LAs which have not responded to the survey. The information gained from our survey will be combined with the data from PHE’s earlier survey and analysed in SPSS (Version 27). This will enable us to understand the different ways in which the Health Check programme is delivered in different contexts and to develop a comprehensive picture of how the policy intent of the programme is translated into practice across England. We anticipate mapping provision against uptake and other elements of the national data set, which will be available at the LA level.

As part of our survey, we will request copies of local evaluations to add to the literature obtained through searching for the realist review. If these local evaluations fulfil our inclusion criteria, they will be included in the review (see step 2 of objective 1 above for more details).

### Objective 3: to provide recommendations

Our programme theory will be used to develop recommendations on tailoring, implementation and design strategies to improve the current delivery and outcomes of the NHS Health Check programme in different settings, for different groups. Further details are provided in the Ethics and Dissemination section.

## Ethics and dissemination

### Dissemination

Our dissemination strategy will build on the participatory approach we have adopted throughout the review process, involving the stakeholders that have engaged with us during the development of this research proposal and throughout the review process. Our approach will be integrative, valuing the different forms of knowledge that are required to produce findings capable of informing complex decision-making.^66^ A range of audiences will be interested in the review’s findings and recommendations, including:

1. Policy-makers, decision-makers and commissioners of NHS Health Checks.
2. Providers of the NHS Health Check and related lifestyle services.
3. Members of the public, including those eligible for NHS Health Checks and relevant advocacy organisations.

Different strategies are likely to be needed for each of these. This project will produce three major types of outputs in addition to the final report:

1. Academic outputs.
2. A range of audience-specific ‘How to’ publications that outline practical advice on tailoring, implementation and design strategies to enhance current NHS Health Check delivery.
3. User-friendly summaries of the review findings tailored to the needs of the different audiences.

We will draw on the advice and expertise within our stakeholder group to help clarify the main ‘players’ for dissemination for each audience and to develop tailored and relevant materials for each group.

### Ethics

Ethical approval for the survey component of our study has been granted by the University of Kent’s SRC Research Ethics Committee (SRCEA ID 0367).

## Supplementary Material

Reviewer comments

Author's manuscript
