# Supplementary material for: NHS Health Check programme: a protocol for a realist review
Source: BMJ Open. 2021 Apr 14;11(4):e048937. doi: 10.1136/bmjopen-2021-048937 (PMC8054100; doi:10.1136/bmjopen-2021-048937)
Supplement: Supplementary data [file bmjopen-2021-048937supp001.pdf]

Supplementary File

Search strategies

The full details of the search strategies for **Step 2a** of our protocol paper is provided below.

MEDLINE (via Ovid)

1	health check*.ti,ab,kw
2	(NHS OR National Health Service OR United Kingdom OR UK OR England OR English).ti,ab,kw
3	exp England/
4	2 or 3
5	1 and 4
6	limit 5 to (english language and yr="2008-Current")

Embase (via Ovid)

1	health check*.ti,ab,kw
2	(NHS OR National Health Service OR United Kingdom OR UK OR England OR English).ti,ab,kw
3	england/
4	2 or 3
5	1 and 4
6	limit 5 to (english language and yr="2008-Current")

CINAHL (via EbscoHost)

S1	TX "health check*"
S2	TI (NHS OR "National Health Service" OR "United Kingdom" OR UK OR England OR English) OR AB (NHS OR "National Health Service" OR "United Kingdom" OR UK OR England OR English)
S3	MH "England"
S4	S2 OR S3
S5	S1 AND S4
S6	Limiters: Published Date: 20080101-20201231; English Language; Expanders: Apply equivalent subjects

HMIC (via Ovid)

1	health check*.mp
2	(NHS OR National Health Service OR United Kingdom OR UK OR England OR English).mp
3	exp england/
4	exp health authorities in england/
5	or/2-4
6	1 and 5
7	limit 6 to (yr="2008-Current")

Web of Science Citation Indexes (SCI-EXPANDED, SSCI)

#1	TOPIC: ("health check*")

#2	TOPIC: (NHS OR "National Health Service" OR "United Kingdom" OR UK OR England OR English)
#3	#1 and #2
#4	Refined by: PUBLICATION YEARS: (2020 OR 2012 OR 2019 OR 2011 OR 2018 OR 2010 OR 2017 OR 2009 OR 2016 OR 2008 OR 2015 OR 2014 OR 2013)<br>AND LANGUAGES: (ENGLISH)
