# Supplementary material for: NHS Health Check programme: a protocol for a realist review
Source: BMJ Open. 2021 Apr 14;11(4):e048937. doi: 10.1136/bmjopen-2021-048937 (PMC8054100; doi:10.1136/bmjopen-2021-048937)
Supplement: Author's manuscript [file bmjopen-2021-048937.draft_revisions.pdf]

BMJ Open

BMJ Open is committed to open peer review. As part of this commitment we make the peer review history of every article we publish publicly available.

When an article is published we post the peer reviewers' comments and the authors' responses online. We also post the versions of the paper that were used during peer review. These are the versions that the peer review comments apply to.

The versions of the paper that follow are the versions that were submitted during the peer review process. They are not the versions of record or the final published versions. They should not be cited or distributed as the published version of this manuscript.

BMJ Open is an open access journal and the full, final, typeset and author-corrected version of record of the manuscript is available on our site with no access controls, subscription charges or pay-per-view fees (http://bmjopen.bmj.com).

If you have any questions on BMJ Open's open peer review process please email info.bmjopen@bmj.com

BMJ Open

NHS Health Check Programme: protocol for a realist review

Journal:	*BMJ Open*
Manuscript ID	bmjopen-2021-048937
Article Type:	Protocol
Date Submitted by the Author:	12-Jan-2021

Complete List of Authors:	Duddy, Claire; Oxford University, Nuffield Department of Primary Care Health Sciences<br>Wong, Geoffrey; Oxford University, Nuffield Department of Primary Care Health Sciences<br>Gadsby, E; University of Kent, Centre for Health Services Studies<br>Krska, Janet; Medway School of Pharmacy, Universities of Greenwich and Kent<br>Hibberd, Vivienne; University of Kent Medway School of Pharmacy, Public Involvement in Pharmacy Studies Group

Keywords:	Risk management < HEALTH SERVICES ADMINISTRATION & MANAGEMENT, PREVENTIVE MEDICINE, PUBLIC HEALTH

SCHOLARONE™
Manuscripts

I, the Submitting Author has the right to grant and does grant on behalf of all authors of the Work (as defined in the below author licence), an exclusive licence and/or a non-exclusive licence for contributions from authors who are: i) UK Crown employees; ii) where BMJ has agreed a CC-BY licence shall apply, and/or iii) in accordance with the terms applicable for US Federal Government officers or employees acting as part of their official duties; on a worldwide, perpetual, irrevocable, royalty-free basis to BMJ Publishing Group Ltd ("BMJ") its licensees and where the relevant Journal is co-owned by BMJ to the co-owners of the Journal, to publish the Work in this journal and any other BMJ products and to exploit all rights, as set out in our licence.

The Submitting Author accepts and understands that any supply made under these terms is made by BMJ to the Submitting Author unless you are acting as an employee on behalf of your employer or a postgraduate student of an affiliated institution which is paying any applicable article publishing charge ("APC") for Open Access articles. Where the Submitting Author wishes to make the Work available on an Open Access basis (and intends to pay the relevant APC), the terms of reuse of such Open Access shall be governed by a Creative Commons licence – details of these licences and which Creative Commons licence will apply to this Work are set out in our licence referred to above.

Other than as permitted in any relevant BMJ Author's Self Archiving Policies, I confirm this Work has not been accepted for publication elsewhere, is not being considered for publication elsewhere and does not duplicate material already published. I confirm all authors consent to publication of this Work and authorise the granting of this licence.

For peer review only

NHS Health Check Programme: protocol for a realist review

Duddy, Claire*
Wong, Geoffrey
Gadsby, Erica
Krska, Janet
Hibberd, Vivienne

***Corresponding author**

Claire Duddy
Nuffield Department of Primary Care Health Sciences
Radcliffe Observatory Quarter
Woodstock Road
Oxford OX2 6GG

claire.duddy@phc.ox.ac.uk
+44 (0)1865 289300

Other authors

Geoff Wong
Nuffield Department of Primary Care Health Sciences, University of Oxford, Oxford, UK

Erica Gadsby
Centre for Health Services Studies, University of Kent, Canterbury CT2 7NF

Janet Krska
Medway School of Pharmacy, The Universities of Greenwich and Kent, Chatham Maritime, Kent ME4 4TB

Vivienne Hibberd (PPI Lead)
Public Involvement in Pharmacy Group, Medway School of Pharmacy, The Universities of Greenwich and Kent, Chatham Maritime, Kent ME4 4TB

Word count (excluding title page, abstract, references, figures, tables): *6829*

For peer review only

Abstract

Introduction

The NHS Health Check aims to identify individuals at increased risk of cardiovascular diseases among the adult population in England. The Health Check includes calculation of CVD risk and discussion of pharmacological and lifestyle approaches to manage risk, including referral to lifestyle support services.

The programme is commissioned by Local Authorities (LAs) and is delivered by a range of different providers in different settings. There is significant variation in activity, with uptake ranging from 25-85% in different areas, and clear evidence of variation in implementation and delivery practice.

Methods and analysis

We aim to understand how the NHS Health Check programme works in different settings, for different groups, so that we can recommend improvements to maximise intended outcomes. To do so, we will undertake a realist review and survey of LA public health teams.

Our review will follow Pawson's five iterative stages: 1) Locate existing theories; 2) Search for evidence; 3) Article selection; 4) Extract and organise data; 5) Synthesise evidence and draw conclusions. Our review will include documents describing local implementation alongside published research studies. We will recruit a stakeholder group (including PHE, commissioners and providers of Health Checks, plus members of the public and patients) to advise us throughout.

Our survey will be sent to all 152 LAs in England to gather detailed information on programme delivery (including Covid-19-related changes) and available referral services. This will enable us to map delivery across England and relate this data to programme outcomes.

Ethics and dissemination

Ethical approval is not required for this review. For the survey, we will seek approval from the University of Kent Research Ethics Committee. Our findings will be used to develop recommendations on tailoring, implementation and design strategies to improve delivery of the NHS Health Check in different settings, for different groups.

Keywords: realist review, survey, NHS Health Check, prevention, risk management, cardiovascular disease

PROSPERO registration: CRD42020163822.

For peer review only

Article summary

Strengths and limitations of this study

- This is the first realist review of the NHS Health Check and will improve our understanding of how the programme works in different settings and for different groups;
- In addition to including published research studies on the Health Check, this review will draw on learning from documents that describe local implementation and innovation in programme delivery;
- Our review will be augmented by a comprehensive survey of local public health teams, capturing new data on current delivery models, including recent innovation in response to the Covid-19 pandemic;
- Our review may be limited by the richness and relevance of the evidence available in the literature;
- Survey response rates may be adversely affected by Covid-19 pressures and we will need to take steps to mitigate these wherever possible.

Background

Cardiovascular disease (CVD) causes a quarter of all deaths in the UK and is the largest cause of premature mortality in deprived areas. Early detection and prevention is an important priority for the NHS in England, and the NHS Long Term Plan (2019) commits to taking wider action on prevention to tackle the underlying causal factors. Over the course of 2020, it has also become clear that many risk factors associated with CVD, and existing health inequalities, are associated with poorer outcomes for Covid-19 patients.(1) The UK government have therefore highlighted the potential role that the NHS Health Check may have to play in helping to address these risk factors.(2)

The NHS Health Check programme is one of the main pillars of CVD prevention efforts in England. It was first launched in 2009, aiming to offer a 5-yearly assessment of individual risk of developing coronary heart disease, stroke, diabetes and chronic renal disease to the population aged between 40 and 74.(3) The Health Check involves measurement of key risk factors and calculation of CVD risk, followed by discussion and agreement on lifestyle and pharmacological approaches to managing the risk. This is a national, mandated programme, originally commissioned by Primary Care Trusts across England. The NHS Health Check programme was re-launched in 2013, when commissioning moved from Primary Care Trusts to Local Authorities (LAs), with an implementation review and action plan.(4) Although tests and measurements are standardised to help ensure the safety, quality and effectiveness of the programme,(5, 6) LAs have flexibility in how and who they commission to provide NHS Health Checks. As commissioning and delivery are determined locally, with the aim of meeting the needs of local populations, there is inevitable variation in delivery, uptake and outcomes.

The total eligible population for the Health Check programme has been estimated to be c. 15.5 million.(7) The largest and most recently published analysis of national data relating to the programme found that almost 10 million eligible people were offered an NHS Heath Check between 2012 and 2017.(8) Of these, 52.6% (just over 5 million) took up the offer. Although national uptake rates generally increased over this period, there was significant regional variation, with uptake rates calculated for upper tier LA areas ranging from 25.1% to 84.7%.(8) These findings are in line with previous analyses that have identified significant variation in invitation and uptake rate for the programme,(9) and variation in Health Check delivery and follow-up, including referrals to lifestyle

For peer review only

services.(10, 11) At present, minimal national data exist to explain these differences, yet it is important to determine what does work, for whom in what setting.

At the time of writing, Public Health England is undertaking a review of the NHS Health Checks, with the aim of making recommendations to improve both the content and delivery of the programme.(12, 13) The review will encompass a wide remit and include consideration of: whether additional 'checks' should be incorporated into the current offering; options to tailor the Health Check and personalise the programme for individuals; and digital tools that may offer opportunities to improve delivery. This review is being undertaken in a wider context of considerable uncertainty for national-level public health functions, following the announcement by Government in August 2020 that Public Health England will be dismantled and a new national institute for health protection created.(14, 15)

Overview of existing evidence

In 2014, Public Health England (PHE) established an Expert Scientific and Clinical Advisory Panel (ESCAP) to continually review the evidence on the NHS Health Check programme.(16) This group recommended periodic syntheses of published evidence. The first such review was a rapid evidence synthesis, published in 2017(11) and updated in 2020.(10) These reviews addressed six research questions identified by PHE, focused on uptake (questions 1-3), management of those at high risk (question 4), patient experience (question 5) and a specific set of outcomes related to the Health Check, including disease detection, referral, reductions in CVD risk and prescribing (question 6). The authors of both reviews identified significant gaps in the literature, and both ESCAP(17) and the review authors made recommendations for action and further research. These recommendations included:

i. Improved characterisation of local variations in implementation of the Health Check, to allow comparisons and sharing of best practice;(11)
ii. The need to develop a model that fully reflects the real-life NHS Health Check intervention and draws on current evidence to estimate its impact;(10, 17)
iii. The need for more research to determine the effect of the Health Check on lifestyle behaviour in different groups.(17)

These rapid evidence syntheses included documents identified by PHE using a systematic and comprehensive search strategy, updated each quarter.(18) Searching was conducted in multiple databases: MEDLINE, PubMed, Embase, HMIC, CINAHL, Global Health, PsycINFO, the Cochrane Library, NHS Evidence, Google Scholar, Google, Clinicaltrials.gov and the ISRCTN registry. The rapid reviews augmented these with additional searches in the Web of Science (Science Citation Index),(10, 11), OpenGrey(11) and a review of abstracts submitted to the 2017 PHE NHS Health Check conference. Together, the two reviews include evidence covering the period January 1996 to December 2019.

In addition to these reviews (and their associated academic publications,(9, 19-21), a further 3 reviews relating to the NHS Health Check have been published to date,(22-24) and we have also identified two other systematic reviews focused on participation and patient experience in similar prevention programmes.(25, 26) Drawing on the existing evidence, below we summarise what is currently known about NHS Health Check programme:

- **Coverage (proportion eligible who receive a Health Check)** is known to vary substantially across regions and settings, but is consistently higher in older people, females and in more deprived populations, although this may reflect targeting.(11) Studies suggest that

For peer review only

community outreach services can reach particular socio-demographic groups,(27) but one study suggests that these services may create inaccuracies in reporting.(28)

- **Uptake (attendance following invitation)** varies across regions and at GP practice level.(24) The evidence on uptake in different groups is highly heterogeneous. There is relatively consistent evidence that older people and women are more likely to take up invitations, but mixed findings in relation to ethnicity and deprivation, with some studies showing higher uptake in specific groups, while others show no difference.(10, 11, 24) There is also clear evidence that uptake is lower amongst smokers.(11, 24).
- **Invitations** are issued in different formats, though letters are most common.(29, 30) Recent studies of the effectiveness of different formats have found that modifications to standard letters, text message invitations/reminders, telephone and opportunistic face-to-face invitations can increase uptake.(10, 11, 29, 31) One cross-sectional study suggests that different invitation methods may be more or less effective for different ethnic and gender groups.(29) Telephone calls including the option to book an appointment during the call may overcome anticipated difficulties in making appointments and offer an opportunity to increase participants' understanding of the Health Check,(32) which may be barriers to uptake.(23) Other barriers may include aversion to preventive medicine, competing priorities and, for community pharmacy and outreach settings, concerns about privacy and confidentiality.(10, 11, 19, 23). Some qualitative evidence highlights the convenience of these settings and the value of community ambassadors.(10, 20)
- **Delivery** of the Health Check varies considerably across settings, despite the standardisation provided by PHE's guidance and the legislation that mandates its delivery. Providers delivering NHS Health Checks have reported challenges with workload, IT, funding, training and the need to cover multiple aspects within one consultation.(10, 11, 23, 33) A recent review found that although many providers recognised the importance of behaviour change to reduce CVD risk, professionals have different views on the contributions of behavioural versus pharmacological interventions, and on the clinical and cost-effectiveness of the Health Check programme.(23) Providers recognise the difficulty patients face in making sustained behaviour and lifestyle changes, acknowledging the need to take patients' social circumstances and resources into account.(34, 35) Professionals have also expressed concerns about limited access to appropriate lifestyle services for onward referrals, and about stretched resources and workload in primary care.(10, 11)
- **Patient experiences** are reported to be positive overall, with patient surveys indicating high levels of 'patient satisfaction' (consistently over 80%) and some reporting that attendance had precipitated lifestyle changes.(10, 11) However, qualitative studies have found that some patients report unmet expectations and confusion around follow up and risk scores. Some patients have found lifestyle advice too simplistic and un-personalised.(10, 11)
- **Outcomes** demonstrating clinical and/or cost effectiveness of the NHS Health Check are harder to obtain. Existing research demonstrates that the Health Check increases the detection of CVD risk factors and disease, and leads to increased statin prescribing (by 3-4%).(10, 11) Some studies also report increased prescribing of antihypertensive drugs (but one cohort study reported that Health Check attendees were *less* likely to receive antihypertensives than matched controls (36)). Three national studies found that the Health Check increased referrals to smoking cessation, weight management, exercise or alcohol support services.(36, 37) However, regional studies demonstrate wide variation in service availability and referral practice across England.(10, 11) The PHE-commissioned rapid reviews identified six primary studies that examined behaviour change, but smoking is the only health behaviour assessed. A limited number of studies have demonstrated post-Health

Check improvements in relevant risk factors, including BMI, diastolic blood pressure, total cholesterol and overall CVD risk, but results across studies are inconsistent and some have found no evidence of any effect.(10)

It is clear across the existing reviews that the emphasis in the literature is on the early steps of the Health Checks pathway, and especially on the invitation to, and uptake of the Health Check. This focus may reflect the variation that is apparent in the published indicators on Health Checks,(7) and emphasis on improving uptake in the legislation that mandates the programme.(38) Studies of Health Check delivery focus on patient and provider experience and perceptions, and there is more limited evidence on what happens after a Health Check. There is a notable absence of studies of post-Health Check behaviour change beyond smoking cessation. At all stages, the existing research demonstrates wide variation in implementation and practice, and significant uncertainties in relation to understanding this variation and the optimal strategies for increasing coverage and uptake, delivery models and maximising important patient outcomes.

Evidence explaining why this research is needed now

Since publication of the rapid evidence review in February 2017, and the rapid review update in 2020, several new studies have been published which add to the findings of the review. A PubMed search conducted in November 2020 for studies published since December 2019 has identified a further 8 empirical studies concerning the NHS Health Check(8, 39-45) and one protocol for an implementation study and trial.(46)

To improve our understanding of how the NHS Health Check achieves its outcomes, it is essential to learn as much as possible from how the programme is delivered, in different settings and by different providers. In the past, case studies have been used by PHE to illustrate 'good practice'.(4) To date, case studies shared by PHE on the Health Checks website (n=24 in November 2020) have focused on sharing practice in relation to increasing coverage or targeting Health Checks invitations to particular groups.(47) Selected local evaluations submitted to PHE have also been shared on the website (n=26 in November 2020), (48) and learning from local implementation of the Health Checks programme has regularly been shared at PHE-run conferences focused on the Health Check and CVD prevention.(49) In addition, a survey of commissioners and providers focused on targeting Health Checks was recently carried out by PHE(50), and a further survey seeking data on local delivery models is currently underway. Both add further local learning which could be used to help understand what works, for whom, how, and in what setting.

None of the reviews conducted to date have effectively utilised this abundant learning from the local level. The first rapid review looked at abstracts submitted to the 2017 NHS Health Check conference, but these form a minor aspect of the review.(11) More recently PHE commissioned the UCL Centre for Behaviour Change to conduct a review of barriers and facilitators to behaviours relevant to NHS Health Checks, including those of providers and invitees/attenders; again, this review included only papers published in academic journals.(23)

It is now eleven years since the Health Checks programme was launched. The amassed evidence from small, local unpublished studies needs to be combined with the published papers, many of which also cover only one locality. This evidence also needs to be combined with more comprehensive knowledge of the variety of ways in which different localities implement the NHS Health Check programme. It is by combining and analysing this evidence that we seek to answer the important research questions set out below.

For peer review only - http://bmjopen.bmj.com/site/about/guidelines.xhtml

Aim and objectives

The NHS Health Check programme is arguably one of the largest prevention programmes of its type in the world, and a cornerstone for the NHS prevention programme. However, many unanswered questions remain. To ensure that our research is sufficiently focussed and will produce findings that are relevant to knowledge users, we sought advice and feedback from colleagues at PHE and our wider stakeholder group to arrive at our aims, objectives and research questions.

Aim

To understand how the NHS Health Check programme works in different settings, for different groups, in order to recommend improvements to maximise intended outcomes.

Objectives

1. To conduct a realist review to enable understanding of how the NHS Health Check programme works in different settings, for different groups to achieve its outcomes.
2. To map how the programme is currently delivered across England, using data collected in a PHE survey (in October 2020), and data we will collect using our own online survey of Local Authorities.
3. To provide recommendations on tailoring, implementation and design strategies to improve the current delivery and outcomes of the NHS Health Check programme in different settings, for different groups.

Review Questions

1. What are the mechanisms by which the current NHS Health Check programme produces its intended outcomes?
2. What are the important contexts which determine whether the different mechanisms produce intended outcomes?
3. In what circumstances are such interventions likely to be effective?

Methods and analysis

Objective 1: To conduct a realist review

The plan of investigation will follow this protocol which is informed by Pawson's five iterative stages in realist reviews (see Figure 1 below).(51) We have chosen to use a realist review approach because the existing research indicates that the NHS Health Check programme is a complex intervention that has a range of outcomes (e.g. variable rates of attendance, follow-up, onwards referral, prescription) which are context sensitive and vary for different groups. We are also aware that the NHS Health Check programme is continually evolving: work exploring the potential for digital services has been under consideration since 2017,(52) and a wide-ranging review of the Health Check programme is currently underway and expected to make recommendations in early 2021.(12) In addition to this, it is clear that the pause and restart of the Health Check programme during the Covid-19 pandemic has provoked a range of responses at local levels, including the introduction of new delivery models in some areas.(53)

Any evidence synthesis that seeks to make sense of how to improve the tailoring, implementation and design of the NHS Health Check programme must take into account the various contexts and interventional strategies in which NHS Health Checks are delivered and the varied outcomes for

For peer review only

different groups. A realist review will be able to generate the knowledge needed to address both these issues.(54) Realist review is an interpretive, theory-driven approach to synthesizing evidence from qualitative, quantitative and mixed-methods research. Its main strength comes from providing findings that coherently and transferably explain how and why context can influence outcomes.

This process of explanation building starts with the development and refinement of a realist 'programme theory' of the NHS Health Check programme. To do this, we have 'mapped' the sequence of steps needed to achieve the final intended outcomes for the programme, taking account of the processes outlined by PHE in their implementation guidance (see Figure 2 below). This initial programme theory will be refined (see Step 1 below) and then further refined and tested against empirical evidence during the review (see Steps 2-5).

Patient and public involvement

Throughout the review, we will consult with a wide range of stakeholders with content expertise and a variety of perspectives. We will invite policy makers, commissioners, training providers, and front-line providers of the Health Checks programme, as well as representatives from relevant charities and members of the public drawn from the eligible population. We will update and extend the membership of this group as needed over the course of the review. We have secured the participation of PHE as a key stakeholder and they have expressed strong interest in assisting us during the review and in our findings. Our initial programme theory will be presented to our stakeholder group and refined based on their feedback. As the programme theory is further refined over the course of the review, the stakeholders will be regularly consulted on the developing findings. The group will meet (either virtually or face to face) four times during the study and communicate via e-mail as necessary. The meetings will be chaired by GW or VH (our PPI Lead, a member from the Public Involvement in Pharmacy Studies at Medway School of Pharmacy group).

This review protocol has been registered with PROSPERO (registration number CRD42020163822) and will follow current quality and publication standards.(55) An overview of the project may be found in **Figure 1** below, and the main steps involved in the review are outlined in more detail below.

[Figure 1]

Step 1: Locate existing theories

The goal of this step is to identify theories that explain how the NHS Health Check programme is supposed to work (and for whom), when it does work, when it does not achieve desired changes in clinical practice and patient outcomes, why it is not effective, and why it is not being used.(56) The rationale for this step is that interventions are "theories incarnate" – that is underpinning the design of such interventions are theories of why certain components are required. In other words, the designers of the programme have put it together in a certain way based on their theories about what needs to be done to get one or more desired outcomes.(57) For example, one theoretical assumption underpinning the programme design is that once patients are given information about their cardiovascular risk score, this knowledge will motivate a proportion of them to make the necessary lifestyle changes to reduce it.(58) There are a number of different theories and models that support such an assumption, though evidence highlights the complexity of achieving sustained behaviour change (e.g.(59)).

To locate relevant theories, we will iteratively:

a) consult with key content experts in our stakeholder group, and

For peer review only - http://bmjopen.bmj.com/site/about/guidelines.xhtml

b) informally search the literature to identify existing theories, including both grey literature in the form of NHS Health Check programme documentation, and published research that has employed formal or substantive theory to understand the programme.

This informal searching differs from the more formal searching process described below in Step 2; it is exploratory and aims to quickly identify a range of possible explanatory theories that may be relevant. Search methods such as citation tracking and snowballing,(60) along with more structured searching for theories(61, 62) will be used.

From these, we will refine an initial programme theory to test in the review. A first version of this initial programme theory is outlined as a starting point in **Figure 2** below. In Step 1, we will refine this model within the project team, and present it to our stakeholder group for their feedback.

[Figure 2]

Focus of the review

To develop this protocol, we undertook searches to identify existing reviews related to the Health Checks programme. In so doing, we identified an existing focus on the early steps in our initial programme theory, and especially on *invitation* and *uptake* of the NHS Health Check (see Figure 2). Conversely, there has been less focus on what happens next, in relation to follow up, onward referral and ongoing support for lifestyle and behaviour change. As a result, we have decided to focus our review efforts on these later steps, which are crucial in delivering the programme's intended outcomes, in relation to supporting individuals to make changes to their behaviour and lifestyle, and ultimately to reduce their risk of experiencing a heart attack, stroke or developing some forms of dementia.(6) We have confirmed the value of this focus with PHE, our key stakeholder, and we will seek further feedback and advice from our wider stakeholder group to help us to develop our thinking about this stage of the Health Checks pathway. However, in recognition of the complex nature of the Health Checks programme, and the possibility that feedback loops may exist, such that the characteristics of one step may be inextricably bound up with others, we will not exclude evidence relating to any other programme steps, but will actively seek out data that might shed light on relationships between each step.

As noted above, we are also aware that the Health Checks programme is continually evolving, and especially aware of the effects of the Covid-19 pandemic on programme delivery, and that a major review of the programme began in August 2020. PHE and the Department of Health and Social Care have acknowledged that many of the factors assessed by the NHS Health Check are also risk factors for severe Covid-19, and the fact that the programme has an explicit aim to reduce health inequalities(2, 13), now a pressing agenda. In addition, restrictions on face-to-face contact, and increased pressure on health services have created a new impetus to adapt programme delivery and may speed up the proposed shift to introduce digital methods or other innovations. Our own review project is taking place within this fast-moving context, and as such, we will aim to prioritise the inclusion of evidence that can support findings and recommendations with relevance for the 'new normal'.

Step 2: Search for evidence

Formal search

The purpose of this Step is to find a relevant 'body of literature' with which to further develop and refine the initial programme theory from Step 1. The search strategy used will be designed, piloted and conducted by CD, in collaboration with the project team. CD is an information specialist with

For peer review only

extensive experience of conducting searches for complex systematic reviews, particularly realist reviews.

At the outset of this project, we were aware that Public Health England regularly undertakes literature searching for new evidence relating to the NHS Health Check. These regular searches employ a very sensitive search strategy across 13 relevant sources (PubMed, MEDLINE, Embase, HMIC, CINAHL, Global Health, PsycINFO, Cochrane Library, NHS Evidence, Google Scholar, Google, Clinicaltrials.gov and the ISRCTN registry).(18) These searches have been used in previous review projects commissioned by PHE to identify evidence relating to the NHS Health Check.(10, 11) These reviews have included additional searches in OpenGrey and/or Web of Science (Science Citation Index); the most recent review captured studies published until the end of December 2019.(10)

We do not intend to duplicate this work, but instead aim to re-use and extend it as follows:

1. We will re-use existing searches by considering for inclusion all documents included in the two existing reviews commissioned by PHE (n=98 documents). These were empirical (quantitative and qualitative) studies of the NHS Health Check. The eligibility criteria employed in each of these reviews is summarised below in **Table 1**.

Table 1: Summary of eligibility criteria for commissioned rapid reviews of the NHS Health Check

**Inclusion criteria**	**Exclusion criteria**
Studies of the NHS Health Check<br>Populations including those eligible for, attending, not attending and providing NHS Health Checks<br>Study designs:<br>RCTs, quasi-RCTs<br>Before and after studies with appropriate comparator groups<br>Interrupted time series<br>Cohort studies (prospective or retrospective)<br>Case-control studies<br>Qualitative studies using recognised methods<br>Economic and health outcome modelling	Editorials, commentaries and opinion pieces

2. We will then seek additional material to consider for inclusion:
    a. We will run additional targeted searches focused on identifying material related to the NHS Health Check that was excluded from the existing reviews. This may include, for example: relevant commentary or opinion, which are not excluded from realist reviews as they may contribute to theory building; studies focused on aspects of the Health Check not covered by the existing reviews; and studies published since December 2019. We will use specific searches to identify additional documents focused on the Health Check programme in England, using specific free text terms describing the Health Check programme alongside relevant subject heading terms as appropriate. We will search MEDLINE, Embase, CINAHL, HMIC and Web of Science (Science and Social Science Citation Indexes).
    b. We will trawl the NHS Health Checks website for documents including case studies (n=24 in November 2020), local evaluations (n=24 in November 2020) and abstracts and posters presented at the Health Checks/Cardiovascular Disease Prevention annual conferences from 2014-2020 (n>450), where these are accessible via current and

For peer review only

archived versions of the website. These sources represent an important source of data on local implementations of the Health Check that has been excluded from previous reviews.

c. For each included published document, we will undertake backwards and forwards citation searches, i.e. for these documents, we will screen their reference lists, and use Google Scholar's 'cited by' links to identify future documents that cited these. Where necessary, we will also undertake further searching to identify 'sibling' documents, related to those that we have already included by virtue of being part of the same broader research projects.(62)

3. If necessary (i.e. if further data is required for programme theory development) we will continue to seek additional material as follows:
   a. We will consider for inclusion all additional studies focused on Health Check (and related international programmes and evidence) included in PHE's published quarterly literature reviews from October 2014 onwards available on the Health Checks website (including those published during our own review project, to ensure the most up to date material was included).
   b. We will run more targeted searches for additional grey literature, including, for example, searching additional relevant websites (Department of Health and Social Care, NHS England, CCGs and LAs).
   c. We will seek access to unpublished evaluation reports by contacting LA public health teams directly (see Objective 2 below for more details).

To ensure we keep up to date with emerging material as the review progresses, we will set up a regular search alert (via Google Scholar) and continue to consult PHE's regularly published literature reviews (via the Health Checks website).

Screening

For the material and searches described above, our inclusion and exclusion criteria are broad as we seek to find quantitative, qualitative and mixed-methods documents, and relevant grey literature. The following criteria will be applied:

Inclusion:

- Intervention: NHS Health Check programme (all delivery models, including face to face and digital)
- Study design: all study designs
- Setting: any setting providing NHS Health Checks in England
- Participants: all adults eligible for NHS Health Checks
- Outcome measures: all outcome measures related to NHS Health Checks

Exclusion:

- Cardiovascular screening programmes run in countries other than England
- Other NHS screening programmes
- Routine health checks offered to specific target populations by the NHS which are not part of the NHS Health Check programme

Screening will be undertaken by CD, based on title and abstract, with a 10% random sample of the citations retrieved from searching will be reviewed independently for quality control by GW. Where

For peer review only

necessary (e.g. no abstract is available) the full text of a document will be consulted. Any disagreements about inclusion will be resolved by discussion. If disagreements remain, the matter will be presented to JK and resolved by majority vote.

Additional searching

An important process in realist reviews is searching for additional data to inform programme theory development. In other words, more searches may be undertaken if we find that we require more data to develop and test certain parts of our programme theory. Some of our proposed strategies for identifying additional data are listed above (point 3), but this list is not necessarily exhaustive. As our programme theory will take into account the wider contextual factors that impact on outcomes from the NHS Health Check, we may also run searches to identify additional data on relevant contexts. For example, we anticipate that we may need to seek evidence relating to the commissioning process, relationships between Health Checks providers (including GPs) and LAs, interprofessional relationships, and the presence of other prevention programmes and related services, which may interact, or overlap with the Health Check.

This additional searching will greatly increase the amount of relevant data available to us for the realist review. For any additional searching undertaken, the project team will discuss and set inclusion and exclusion criteria. CD will develop, pilot and refine additional search strategies as needed. Screening will be conducted as described above. As in Step 1, these searches are likely to be exploratory and purposive, potentially seeking documents from a wide range of disciplines. Where applicable, we will follow search strategies described by Booth et al, developed for just such data.(62)

Step 3: Article selection

Following initial screening, documents will be read in full text and selected for inclusion in the review based on an assessment of *relevance* (whether data can contribute to theory building and/or testing) and *rigour* (whether the methods used to generate the relevant data are credible and trustworthy).(63) Even when a document found from the initial search has been screened and has met the inclusion criteria, it may still not contain any data that is relevant for programme theory development and refinement.

CD will read the full text of all the documents that have been included after initial screening. Documents will be selected for inclusion when they contain data that is relevant to the realist analysis – i.e. could inform some aspect of the programme theory. At the point of inclusion based on relevance, an assessment will also be made of rigour (how trustworthy were methods used to generate the data). To illustrate how we will operationalise the assessment of rigour: if data have been generated using a questionnaire/survey, the trustworthiness of the data will be considered to be higher if the questionnaire had previously been shown to be reliable and valid, and remained unaltered (or where subsequent testing had been undertaken following any alterations). However, data may still be included even if judged to be of limited rigour, as we will also make an overall assessment of rigour at the level of the programme theory.(64) In other words, we will also consider the role that each piece of data plays in our developing programme theory and how it strengthens (or not) our explanations of outcomes.

A random sample of 10% of documents identified as including relevant data will be selected, assessed and discussed between CD and GW to ensure that decisions to finally include have been made consistently. The remaining 90% of decisions will be made by CD (though a number of these may require further discussion and joint reading within the project team, where there is any

For peer review only

uncertainty over issues of relevance or rigour. As necessary, we will employ the same decision-making processes as were used during screening in Step 2.

Step 4: Extracting and organising data

The main characteristics (bibliographic details and information relating to study design, participants, settings and findings) of the included documents will be extracted into an Excel spreadsheet.

The full text of included documents will be uploaded into NVivo (a qualitative data analysis software tool). Relevant sections of text in these documents will be coded in NVivo, and interpreted as relating to contexts, mechanisms and outcomes, or relationships between these. Coding will be both deductive (codes created in advance of data extraction and analysis will be informed by the initial programme theory), inductive (codes will be created to categorise data reported in included studies), and retroductive (codes will be created based on an interpretation of data, to infer the causal forces that generate observed outcomes, i.e. mechanisms). Each new element of data will be used to refine the theory if appropriate, and as the theory is refined, included studies will be re-scrutinised to search for data relevant to the revised theory that may have been missed.

Data extraction and organisation will be undertaken by CD. As with screening and inclusion decisions, a random sample of 10% of documents will be independently checked by GW for quality control. Any disagreements will be resolved by discussion and if disagreements remain JK will be asked for her opinion, and resolution will be by majority vote.

Step 5: Synthesising the evidence and drawing conclusions

We will use a realist logic of analysis to make sense of data included in the review. CD will undertake this step with support from GW and EG. We will use a series of questions about the relevance and rigour of content within data sources as part of our process of analysis and synthesis(65):

- Relevance: Are sections of text within this document relevant to programme theory development?
- Rigour (judgements about trustworthiness): Are these data sufficiently trustworthy to warrant making changes to any aspect of the programme theory?
- Interpretation of meaning: If the section of text is relevant and trustworthy enough, do its contents provide data that may be interpreted as functioning as context, mechanism or outcome?
- Interpretations and judgements about Context-Mechanism-Outcome-Configurations (CMOCs): What is the CMOC (partial or complete) for the data that has been interpreted as functioning as context, mechanism or outcome? Are there further data to inform the particular CMOCs contained within this document or other documents? If so, which other documents? How does this particular CMOC relate to other CMOCs that have already been developed?
- Interpretations and judgements about programme theory: How does this particular (full or partial) CMOC relate to the programme theory? Within this same document are there data which inform how the CMOC relates to the programme theory? If not, are there data in other documents? Which ones? In light of this particular CMOC and any supporting data, does the programme theory need to be changed?

Data to inform our interpretation of the relationships between contexts, mechanisms and outcomes will be sought not just within the same document, but across documents (e.g. mechanisms inferred from one document could help explain the way contexts influenced outcomes in a different

For peer review only

document). Synthesising data from different documents is often necessary to compile CMOCs, since not all parts of the configurations will always be articulated in the same document.

Within the analytic process set out above, we will use interpretive cross-case comparison to understand and explain how and why reported outcomes have occurred, for example, by comparing the elements within the NHS Health Check programme which have produced a particular outcome against those which have not, to understand how context has influenced reported findings. When working through the questions set out, where appropriate we will use the following forms of reasoning to make sense of the data:

- Juxtaposition of data: for example, where data about how Health Checks setting influenced outcome in one document enables insights into data about outcomes in another document.
- Reconciling of data: where data differ in apparently similar circumstances, further investigation is appropriate in order to find explanations for why these differences have occurred.
- Adjudication of data: where there are conflicting data, plausibility of these data can be informed on the basis of methodological strengths or weaknesses of the data collection methods.
- Consolidation of data: where outcomes differ in particular contexts, an explanation can be constructed of how and why these outcomes occur differently.

In addition to the material identified for inclusion in the review, additional information obtained via a survey will also provide rich data on contexts and mechanisms in different localities, which will enhance our ability to make sense of the data from the documents identified in the searches. See Objective 2 below for more details.

Objective 2: To map current delivery across England

This objective will both enable additional material (local knowledge, unpublished evaluations and examples of best practice and Covid-related innovation) to be identified for the review and provide a comprehensive overview of how different localities across England implement the NHS Health Check programme. It will be conducted (with support from the project team and stakeholder group) by EG, a senior public health researcher with a strong understanding of LA commissioning and public health service provision.

PHE conducted a survey of LA commissioners in October 2020 as part of the wider review of the Health Checks programme. From this survey, they have provided detailed information from the 104 responding authorities related to some aspects of the programme's delivery from April 2019 until March 2020, in particular: how the eligible population was identified; what methods were used for first invitation; when and in which settings Health Check appointments were made available, and who provided them; whether and what type of point of care testing was used; whether digital solutions were used in delivery; and how the provider workforce was supported. PHE and their survey respondents have granted us permission to include these data in our study.

We will supplement this existing information with our own survey, by asking questions related to current delivery models (in 2021, following the Covid-19 related pause to the service) and questions related to options for onward referral and follow-up of patients after the Health Check encounter. In particular, our survey will identify the extent to which commissioners and providers are changing the way they commission and deliver the NHS Health Check programme in light of the Covid-19 pandemic. We will also identify the extent to which services are available to support those identified as having modifiable risk factors, which will help us to address our review focus on what happens

For peer review only - http://bmjopen.bmj.com/site/about/guidelines.xhtml

after a Health Check, especially in relation to follow up, onward referral and ongoing support for lifestyle and behaviour change.

Our survey will be designed in collaboration with our stakeholders and pilot tested prior to being distributed as an online survey (using Jisc Online Surveys) to all 152 upper-tier and unitary LAs in England. To ensure a maximal response, and to ensure the survey is correctly targeted to those who can answer it, we will work closely with PHE to distribute the survey and make use of their tried-and-tested processes for dissemination. PHE have agreed to send the survey on our behalf through their local networks and send up to two reminders to non-responders. They will also publicise it through their established national and local communications channels (including the NHS Health Check e-Bulletin, website and Twitter feed).

Survey responses will be logged, managed and sorted for analysis using Microsoft Excel. Where there are missing data from completed surveys, we will search each LA's website to see if we can find the necessary information. Resources and time permitting, we will also search for data from the websites of LAs which have not responded to the survey. The information gained from our survey will be combined with the data from PHE's earlier survey and analysed in SPSS. This will enable us to understand the different ways in which the Health Checks programme is delivered in different contexts and to develop a comprehensive picture of how the policy intent of the programme is translated into practice across England. We anticipate mapping provision against uptake and other elements of the national dataset, which will be available at LA level.

As part of our survey, we will request copies of local evaluations to add to the literature obtained through searching for the realist review. If these local evaluations fulfil our inclusion criteria they will be included in the review (see Step 2 of Objective 1 above for more details).

Objective 3: To provide recommendations

Our programme theory will be used to develop recommendations on tailoring, implementation and design strategies to improve the current delivery and outcomes of the NHS Health Checks programme in different settings, for different groups. Further details are provided in the Ethics and Dissemination section below.

Ethics and dissemination

Dissemination

Our dissemination strategy will build on the participatory approach we have adopted throughout the review process, involving the stakeholders that have engaged with us during the development of this research proposal and throughout the review process. Our approach will be integrative, valuing the different forms of knowledge that are required to produce findings capable of informing complex decision making.(66) A range of audiences will be interested in the review's findings and recommendations, including:

a. Policy makers, decision-makers and commissioners of NHS Health Checks;
b. Providers of the NHS Health Check and related lifestyle services;
c. Members of the public, including those eligible for NHS Health Checks and relevant advocacy organisations

Different strategies are likely to be needed for each of these. This project will produce three major types of outputs in addition to the final report:

For peer review only

a. Academic outputs;
b. A range of audience-specific ‘How to’ publications that outline practical advice on tailoring, implementation and design strategies to enhance current NHS Health Check delivery;
c. User-friendly summaries of the review findings tailored to the needs of the different audiences.

We will draw on the advice and expertise within our stakeholder group to help clarify the main ‘players’ for dissemination for each audience, and to develop tailored and relevant materials for each group.

Ethics

We will seek approval from the University of Kent Research Ethics Committee for the survey.

For peer review only

Author contributions

GW and JK conceived the study with input from EG and CD. GW and JK led the design and wrote the first draft of this protocol and JK, EG, CD and VH provided criticism and refinement. CD designed the search strategy. All the authors have read and approved the final manuscript.

Funding statement

This project is funded by the National Institute for Health Research (NIHR) Health Services and Delivery Research Programme (Grant Reference Number NIHR 129209). The views expressed in this publication are those of the author(s) and not necessarily those of the NIHR or the Department of Health and Social Care.

Competing interests

CD and GW are both members of the Royal College of General Practitioners (United Kingdom) Overdiagnosis and Overtreatment Group.

GW is an NHS General Practitioner and deputy chair of the National Institute for Health Research Health Technology Assessment Prioritisation Committee: Integrated Community Health and Social Care Panel (A).

Figure legends

Figure 1: Project flow diagram

Figure 2: Initial programme theory behind NHS Health Checks and some of the processes that may influence outcomes

For peer review only

References

1. NHS. Who's at higher risk from coronavirus 2020 [Available from: https://www.nhs.uk/conditions/coronavirus-covid-19/people-at-higher-risk/whos-at-higher-risk-from-coronavirus/.
2. Cabinet Office. Our plan to rebuild: The UK Government's COVID-19 recovery strategy. 2020.
3. NHS. NHS Health Check 2020 [Available from: https://www.nhs.uk/conditions/nhs-health-check/.
4. Public Health England. NHS Health Check implementation review and action plan Summary. 2013.
5. Public Health England. NHS Health Check programme standards: a framework for quality improvement July 2020. 2020.
6. Public Health England. NHS Health Check Best practice guidance for commissioners and providers October 2019. 2019.
7. Public Health England. NHS Health Checks Fingertips Profile 2020 [Available from: https://fingertips.phe.org.uk/profile/nhs-health-check-detailed.
8. Patel R, Barnard S, Thompson K, Lagord C, Clegg E, Worrall R, et al. Evaluation of the uptake and delivery of the NHS Health Check programme in England, using primary care data from 9.5 million people: a cross-sectional study. BMJ Open. 2020;10(11):e042963.
9. Martin A, Saunders CL, Harte E, Griffin SJ, MacLure C, Mant J, et al. Delivery and impact of the NHS Health Check in the first 8 years: a systematic review. Br J Gen Pract. 2018;68(672):e449-e59.
10. Tanner L, Kenny R, Still M, Pearson F, Bhardwaj-Gosling R. NHS Health Check Programme Rapid Review Update. University of Sunderland, Newcastle University, Public Health England; 2020.
11. Usher-Smith JA, Mant J, Martin A, Harte E, MacLure C, Meads C, et al. NHS Health Check Programme rapid evidence synthesis. Cambridge, UK: The Primary Care Unit, University of Cambridge, RAND Europe, Public Health England; 2017.
12. Cabinet Office, Department of Health and Social Care. Advancing our health: prevention in the 2020s - consultation document. 2019.
13. Public Health England. NHS Health Check e-Bulletin - August 2020 2020 [Available from: https://www.nhshealthcheck.nhs.uk/nhs-health-check-e-bulletin-august-2020/front-page/nhs-health-check-e-bulletin-august-2020.
14. Government creates new National Institute for Health Protection [press release]. 18 August 2020 2020.
15. Department of Health & Social Care. The future of public health: the National Institute for Health Protection and other public health functions. 2020 15 September 2020.
16. Public Health England. NHS Health Check: our approach to the evidence. 2013 July 2013.
17. Public Health England. Emerging evidence on the NHS Health Check: findings and recommendations. A report from the Expert Scientific and Clinical Advisory Panel. 2017.
18. Public Health England. NHS Health Check: Literature Review 2020 [Available from: https://www.healthcheck.nhs.uk/commissioners-and-providers/evidence/literature-review/.
19. Harte E, MacLure C, Martin A, Saunders CL, Meads C, Walter FM, et al. Reasons why people do not attend NHS Health Checks: a systematic review and qualitative synthesis. Br J Gen Pract. 2018;68(666):e28-e35.
20. Usher-Smith JA, Harte E, MacLure C, Martin A, Saunders CL, Meads C, et al. Patient experience of NHS health checks: a systematic review and qualitative synthesis. BMJ Open. 2017;7(8):e017169.
21. Mills K, Harte E, Martin A, MacLure C, Griffin SJ, Mant J, et al. Views of commissioners, managers and healthcare professionals on the NHS Health Check programme: a systematic review. BMJ Open. 2017;7(11):e018606.

For peer review only

22. Cooper A, Dugdill L. Evidence of improved uptake of health checks: rapid review. University of Salford,; 2014.
23. Atkins L, Stefanidou C, Chadborn T, Thompson K, Michie S, Lorencatto F. Influences on NHS Health Check behaviours: a systematic review. BMC Public Health. 2020;20(1):1359.
24. Bunten A, Porter L, Gold N, Bogle V. A systematic review of factors influencing NHS health check uptake: invitation methods, patient characteristics, and the impact of interventions. BMC Public Health. 2020;20(1):93.
25. Shaw RL, Holland C, Pattison HM, Cooke R. Patients' perceptions and experiences of cardiovascular disease and diabetes prevention programmes: A systematic review and framework synthesis using the Theoretical Domains Framework. Soc Sci Med. 2016;156:192-203.
26. de Waard AM, Wandell PE, Holzmann MJ, Korevaar JC, Hollander M, Gornitzki C, et al. Barriers and facilitators to participation in a health check for cardiometabolic diseases in primary care: A systematic review. Eur J Prev Cardiol. 2018;25(12):1326-40.
27. Woringer M, Cecil E, Watt H, Chang K, Hamid F, Khunti K, et al. Evaluation of community provision of a preventive cardiovascular programme - the National Health Service Health Check in reaching the under-served groups by primary care in England: cross sectional observational study. BMC Health Serv Res. 2017;17(1):405.
28. Riley VA, Gidlow C, Ellis NJ. Uptake of NHS health check: issues in monitoring. Prim Health Care Res Dev. 2018:1-4.
29. Cook EJ, Sharp C, Randhawa G, Guppy A, Gangotra R, Cox J. Who uses NHS health checks? Investigating the impact of ethnicity and gender and method of invitation on uptake of NHS health checks. Int J Equity Health. 2016;15:13.
30. Gidlow C, Ellis N, Randall J, Cowap L, Smith G, Iqbal Z, et al. Method of invitation and geographical proximity as predictors of NHS Health Check uptake. Journal of public health (Oxford, England). 2015;37(2):195-201.
31. Mason A, Liu D, Marks L, Davis H, Hunter D, Jehu LM, et al. Local authority commissioning of NHS Health Checks: A regression analysis of the first three years. Health Policy. 2018;122(9):1035-42.
32. Brangan E, Stone TJ, Chappell A, Harrison V, Horwood J. Patient experiences of telephone outreach to enhance uptake of NHS Health Checks in more deprived communities and minority ethnic groups: A qualitative interview study. Health Expect. 2019;22(3):364-72.
33. Chatterjee R, Chapman T, Brannan MG, Varney J. GPs' knowledge, use, and confidence in national physical activity and health guidelines and tools: a questionnaire-based survey of general practice in England. Br J Gen Pract. 2017;67(663):e668-e75.
34. Alageel S, Gulliford MC, McDermott L, Wright AJ. Implementing multiple health behaviour change interventions for cardiovascular risk reduction in primary care: a qualitative study. BMC Fam Pract. 2018;19(1):171.
35. Ismail H, Atkin K. The NHS Health Check programme: insights from a qualitative study of patients. Health Expectations. 2016;19(2):345-55.
36. Alageel S, Gulliford MC. Health checks and cardiovascular risk factor values over six years' follow-up: Matched cohort study using electronic health records in England. PLoS Med. 2019;16(7):e1002863.
37. Robson J, Dostal I, Sheikh A, Eldridge S, Madurasinghe V, Griffiths C, et al. The NHS Health Check in England: an evaluation of the first 4 years. BMJ Open. 2016;6(1):e008840.
38. The Local Authorities (Public Health Functions and Entry to Premises by Local Healthwatch Representatives) Regulations, Stat. 351 (2013).
39. Sallis A, Gold N, Agbebiyi A, James RJE, Berry D, Bonus A, et al. Increasing uptake of National Health Service Health Checks in primary care: a pragmatic randomized controlled trial of enhanced invitation letters in Northamptonshire, England. J Public Health (Oxf). 2019.
40. Alageel S, Gulliford MC, Wright A, Khoshaba B, Burgess C. Engagement with advice to reduce cardiovascular risk following a health check programme: A qualitative study. Health Expect. 2020;23(1):193-201.

For peer review only

41. Chattopadhyay K, Biswas M, Moore R. NHS Health Check and healthy lifestyle in Leicester, England: analysis of a survey dataset. Perspect Public Health. 2020;140(1):27-37.
42. Gidlow CJ, Ellis NJ, Cowap L, Riley VA, Crone D, Cottrell E, et al. Quantitative examination of video-recorded NHS Health Checks: comparison of the use of QRISK2 versus JBS3 cardiovascular risk calculators. BMJ Open. 2020;10(9):e037790.
43. Hardeman W, Mitchell J, Pears S, Van Emmenis M, Theil F, Gc VS, et al. Evaluation of a very brief pedometer-based physical activity intervention delivered in NHS Health Checks in England: The VBI randomised controlled trial. PLoS Med. 2020;17(3):e1003046.
44. Palladino R, Vamos EP, Chang KC, Khunti K, Majeed A, Millett C. Evaluation of the Diabetes Screening Component of a National Cardiovascular Risk Assessment Programme in England: a Retrospective Cohort Study. Sci Rep. 2020;10(1):1231.
45. Paxton B, Mills K, Usher-Smith JA. Fidelity of the delivery of NHS Health Checks in general practice: an observational study. BJGP Open. 2020;4(4).
46. Nahar P, van Marwijk H, Gibson L, Musinguzi G, Anthierens S, Ford E, et al. A protocol paper: community engagement interventions for cardiovascular disease prevention in socially disadvantaged populations in the UK: an implementation research study. Glob Health Res Policy. 2020;5:12.
47. Public Health England. NHS Health Checks - Case Studies 2020 [Available from: https://www.healthcheck.nhs.uk/commissioners-and-providers/evidence/case-studies/.
48. Public Health England. NHS Health Check - Local evaluations 2020 [Available from: https://www.healthcheck.nhs.uk/commissioners-and-providers/evidence/local-evaluation/.
49. Public Health England. NHS Health Checks - Webinars and Conferences 2020 [Available from: https://www.healthcheck.nhs.uk/commissioners-and-providers/events/.
50. England PH. NHS Health Check commissioning: Review of commissioner's current and potential use of weighted financial remuneration. 2018.
51. Pawson R, Greenhalgh T, Harvey G, Walshe K. Realist review - a new method of systematic review designed for complex policy interventions. Journal of Health Services Research & Policy. 2005;10(1_suppl):21-34.
52. Public Health England. NHS Health Checks - NHS Health Check Programme Digital Exemplar 2020 [Available from: https://www.healthcheck.nhs.uk/nhs-health-check-digital-exemplar/.
53. Public Health England. NHS Health Check: Restart Preparation [Webinar]. 2020.
54. Wong G. Is complexity just too complex? J Clin Epidemiol. 2013;66(11):1199-201.
55. Wong G, Greenhalgh T, Westhorp G, Buckingham J, Pawson R. RAMESES publication standards: realist syntheses. BMC Medicine. 2013;11(1):21.
56. Pawson R, Owen L, Wong G. The Today Programme's Contribution to Evidence-based Policy. Evaluation. 2010;16(2):211-3.
57. Pawson R. Realist Methodology: The Building Blocks of Evidence. 2006 2020/11/10. In: Evidence-based policy: A realist perspective [Internet]. London: SAGE Publications Ltd. Available from: https://methods.sagepub.com/book/evidence-based-policy.
58. Department of Health. Economic Modelling for Vascular Checks: A technical consultation on the work undertaken to establish the clinical and cost effectiveness evidence base for the Department of Health's policy of vascular checks. In: Health. Do, editor. 2008.
59. Michie S, Atkins L, West R. The behaviour change wheel: a guide to designing interventions. London: Silverback Publishing; 2014.
60. Greenhalgh T, Peacock R. Effectiveness and efficiency of search methods in systematic reviews of complex evidence: audit of primary sources. BMJ. 2005;331(7524):1064-5.
61. Booth A, Carroll C. Systematic searching for theory to inform systematic reviews: is it feasible? Is it desirable? Health Information & Libraries Journal. 2015;32(3):220-35.
62. Booth A, Harris J, Croot E, Springett J, Campbell F, Wilkins E. Towards a methodology for cluster searching to provide conceptual and contextual "richness" for systematic reviews of complex interventions: case study (CLUSTER). BMC Medical Research Methodology. 2013;13(1):118.

For peer review only

63. Pawson R. Realist synthesis: new protocols for systematic review. 2006. In: Evidence-based policy: a realist perspective [Internet]. [73-104].
64. Wong G. Data gathering in realist reviews: Looking for needles in haystacks. 2018. In: Doing Realist Research [Internet]. London: SAGE Publications Ltd; [131-46].
65. Papoutsi C, Mattick K, Pearson M, Brennan N, Briscoe S, Wong G. Interventions to improve antimicrobial prescribing of doctors in training (IMPACT): a realist review. NIHR Health Services and Delivery Research,. 2018.
66. Bowen S, Graham I. Integrated knowledge translation. 2013. In: Knowledge Translation in Health Care [Internet]. John Wiley & Sons; [14-23].

For peer review only

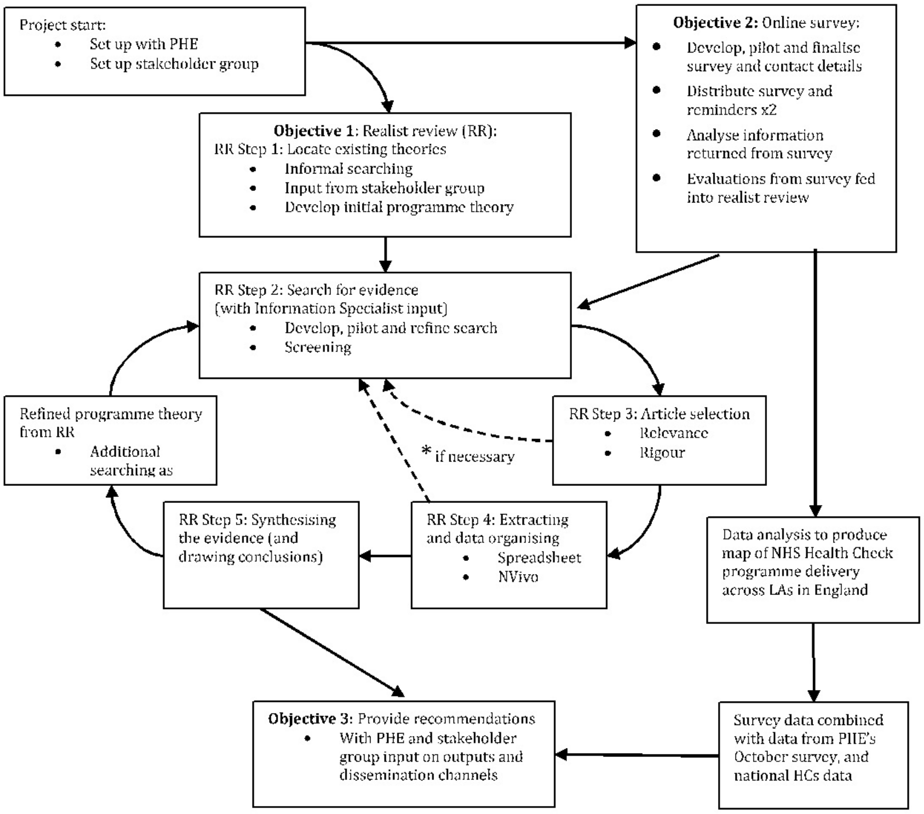

Figure 1: Project flow diagram

248x220mm (120 x 120 DPI)

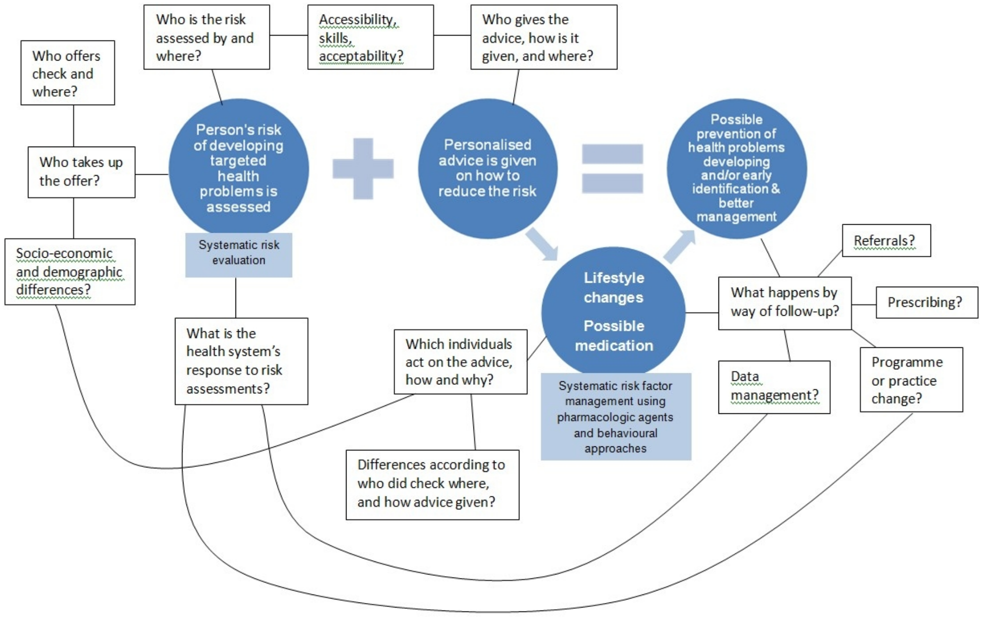

Figure 2: Initial programme theory behind NHS Health Checks and some of the processes that may influence outcomes

296x188mm (96 x 96 DPI)

Reporting checklist for protocol of a systematic review.

Based on the PRISMA-P guidelines.

Instructions to authors

Complete this checklist by entering the page numbers from your manuscript where readers will find each of the items listed below.

Your article may not currently address all the items on the checklist. Please modify your text to include the missing information. If you are certain that an item does not apply, please write "n/a" and provide a short explanation.

Upload your completed checklist as an extra file when you submit to a journal.

In your methods section, say that you used the PRISMA-Preporting guidelines, and cite them as:

Moher D, Shamseer L, Clarke M, Ghersi D, Liberati A, Petticrew M, Shekelle P, Stewart LA. Preferred Reporting Items for Systematic Review and Meta-Analysis Protocols (PRISMA-P) 2015 statement. Syst Rev. 2015;4(1):1.

		Reporting Item	Page Number
**Title**			
Identification	#1a	Identify the report as a protocol of a systematic review	1
Update	#1b	If the protocol is for an update of a previous systematic review, identify as such	n/a
**Registration**			
	#2	If registered, provide the name of the registry (such as PROSPERO) and registration number	2
**Authors**			
Contact	#3a	Provide name, institutional affiliation, e-mail address of all protocol authors; provide physical mailing address of corresponding author	1
Contribution	#3b	Describe contributions of protocol authors and identify the guarantor of the review	16
**Amendments**			

For peer review only

	#4	If the protocol represents an amendment of a previously completed or published protocol, identify as such and list changes; otherwise, state plan for documenting important protocol amendments	n/a
**Support**			
Sources	#5a	Indicate sources of financial or other support for the review	16
Sponsor	#5b	Provide name for the review funder and / or sponsor	16
Role of sponsor or funder	#5c	Describe roles of funder(s), sponsor(s), and / or institution(s), if any, in developing the protocol	n/a
**Introduction**			
Rationale	#6	Describe the rationale for the review in the context of what is already known	3-7
Objectives	#7	Provide an explicit statement of the question(s) the review will address with reference to participants, interventions, comparators, and outcomes (PICO)	8
**Methods**			
Eligibility criteria	#8	Specify the study characteristics (such as PICO, study design, setting, time frame) and report characteristics (such as years considered, language, publication status) to be used as criteria for eligibility for the review	12
Information sources	#9	Describe all intended information sources (such as electronic databases, contact with study authors, trial registers or other grey literature sources) with planned dates of coverage	9-10
Search strategy	#10	Present draft of search strategy to be used for at least one electronic database, including planned limits, such that it could be repeated	n/a - not yet developed
Study records - data management	#11a	Describe the mechanism(s) that will be used to manage records and data throughout the review	12-13
Study records - selection process	#11b	State the process that will be used for selecting studies (such as two independent reviewers) through each	12

For peer review only

		phase of the review (that is, screening, eligibility and inclusion in meta-analysis)	
Study records - data collection process	#11c	Describe planned method of extracting data from reports (such as piloting forms, done independently, in duplicate), any processes for obtaining and confirming data from investigators	13
Data items	#12	List and define all variables for which data will be sought (such as PICO items, funding sources), any pre-planned data assumptions and simplifications	13
Outcomes and prioritization	#13	List and define all outcomes for which data will be sought, including prioritization of main and additional outcomes, with rationale	13
Risk of bias in individual studies	#14	Describe anticipated methods for assessing risk of bias of individual studies, including whether this will be done at the outcome or study level, or both; state how this information will be used in data synthesis	n/a. Relevance and rigour will be assessed as is appropriate for realist review (see page 13)
Data synthesis	#15a	Describe criteria under which study data will be quantitatively synthesised	n/a
Data synthesis	#15b	If data are appropriate for quantitative synthesis, describe planned summary measures, methods of handling data and methods of combining data from studies, including any planned exploration of consistency (such as I2, Kendall's τ)	n/a
Data synthesis	#15c	Describe any proposed additional analyses (such as sensitivity or subgroup analyses, meta-regression)	n/a
Data synthesis	#15d	If quantitative synthesis is not appropriate, describe the type of summary planned	13-14
Meta-bias(es)	#16	Specify any planned assessment of meta-bias(es) (such as publication bias across studies, selective reporting within studies)	n/a
Confidence in cumulative evidence	#17	Describe how the strength of the body of evidence will be assessed (such as GRADE)	n/a

Notes:

For peer review only

- 10: n/a - not yet developed
- 14: n/a. Relevance and rigour will be assessed as is appropriate for realist review (see page 13) The PRISMA-P checklist is distributed under the terms of the Creative Commons Attribution License CC-BY 4.0. This checklist was completed on 11. January 2021 using https://www.goodreports.org/, a tool made by the EQUATOR Network in collaboration with Penelope.ai

For peer review only

BMJ Open

NHS Health Check Programme: protocol for a realist review

Journal:	*BMJ Open*
Manuscript ID	bmjopen-2021-048937.R1
Article Type:	Protocol
Date Submitted by the Author:	27-Feb-2021
Complete List of Authors:	Duddy, Claire; Oxford University, Nuffield Department of Primary Care Health Sciences<br>Wong, Geoff; Oxford University, Nuffield Department of Primary Care Health Sciences<br>Gadsby, E; University of Kent, Centre for Health Services Studies<br>Krska, Janet; Medway School of Pharmacy, Universities of Greenwich and Kent<br>Hibberd, Vivienne; University of Kent Medway School of Pharmacy, Public Involvement in Pharmacy Studies Group
**Primary Subject Heading**:	Public health
Secondary Subject Heading:	Cardiovascular medicine, Health policy
Keywords:	Risk management < HEALTH SERVICES ADMINISTRATION & MANAGEMENT, PREVENTIVE MEDICINE, PUBLIC HEALTH

SCHOLARONE™
Manuscripts

I, the Submitting Author has the right to grant and does grant on behalf of all authors of the Work (as defined in the below author licence), an exclusive licence and/or a non-exclusive licence for contributions from authors who are: i) UK Crown employees; ii) where BMJ has agreed a CC-BY licence shall apply, and/or iii) in accordance with the terms applicable for US Federal Government officers or employees acting as part of their official duties; on a worldwide, perpetual, irrevocable, royalty-free basis to BMJ Publishing Group Ltd ("BMJ") its licensees and where the relevant Journal is co-owned by BMJ to the co-owners of the Journal, to publish the Work in this journal and any other BMJ products and to exploit all rights, as set out in our licence.

The Submitting Author accepts and understands that any supply made under these terms is made by BMJ to the Submitting Author unless you are acting as an employee on behalf of your employer or a postgraduate student of an affiliated institution which is paying any applicable article publishing charge ("APC") for Open Access articles. Where the Submitting Author wishes to make the Work available on an Open Access basis (and intends to pay the relevant APC), the terms of reuse of such Open Access shall be governed by a Creative Commons licence – details of these licences and which Creative Commons licence will apply to this Work are set out in our licence referred to above.

Other than as permitted in any relevant BMJ Author's Self Archiving Policies, I confirm this Work has not been accepted for publication elsewhere, is not being considered for publication elsewhere and does not duplicate material already published. I confirm all authors consent to publication of this Work and authorise the granting of this licence.

For peer review only

NHS Health Check Programme: protocol for a realist review

Duddy, Claire*
Wong, Geoffrey
Gadsby, E W
Krska, Janet
Hibberd, Vivienne

***Corresponding author**

Claire Duddy
Nuffield Department of Primary Care Health Sciences
Radcliffe Observatory Quarter
Woodstock Road
Oxford OX2 6GG

claire.duddy@phc.ox.ac.uk
+44 (0)1865 289300

Other authors

Geoff Wong
Nuffield Department of Primary Care Health Sciences, University of Oxford, Oxford, UK

Erica Gadsby
Centre for Health Services Studies, University of Kent, Canterbury CT2 7NF

Janet Krska
Medway School of Pharmacy, The Universities of Greenwich and Kent, Chatham Maritime, Kent ME4 4TB

Vivienne Hibberd (PPI Lead)
Public Involvement in Pharmacy Group, Medway School of Pharmacy, The Universities of Greenwich and Kent, Chatham Maritime, Kent ME4 4TB

Word count (excluding title page, abstract, references, figures, tables): *6829*

For peer review only

Abstract

Introduction

The NHS Health Check aims to identify individuals at increased risk of cardiovascular diseases among the adult population in England. The Health Check includes calculation of CVD risk and discussion of pharmacological and lifestyle approaches to manage risk, including referral to lifestyle support services.

The programme is commissioned by Local Authorities (LAs) and is delivered by a range of different providers in different settings. There is significant variation in activity, with uptake ranging from 25-85% in different areas, and clear evidence of variation in implementation and delivery practice.

Methods and analysis

We aim to understand how the NHS Health Check programme works in different settings, for different groups, so that we can recommend improvements to maximise intended outcomes. To do so, we will undertake a realist review and survey of LA public health teams.

Our review will follow Pawson's five iterative stages: 1) Locate existing theories; 2) Search for evidence; 3) Article selection; 4) Extract and organise data; 5) Synthesise evidence and draw conclusions. Our review will include documents describing local implementation alongside published research studies. We will recruit a stakeholder group (including PHE, commissioners and providers of Health Checks, plus members of the public and patients) to advise us throughout.

Our survey will be sent to all 152 LAs in England to gather detailed information on programme delivery (including Covid-19-related changes) and available referral services. This will enable us to map delivery across England and relate this data to programme outcomes.

Ethics and dissemination

Ethical approval is not required for this review. For the survey, we will seek approval from the University of Kent Research Ethics Committee. Our findings will be used to develop recommendations on tailoring, implementation and design strategies to improve delivery of the NHS Health Check in different settings, for different groups.

Keywords: realist review, survey, NHS Health Check, prevention, risk management, cardiovascular disease

PROSPERO registration: CRD42020163822.

For peer review only

Article summary

Strengths and limitations of this study

- This is the first realist review of the NHS Health Check and will improve our understanding of how the programme works in different settings and for different groups;
- In addition to including published research studies on the Health Check, this review will draw on learning from documents that describe local implementation and innovation in programme delivery;
- Our review will be augmented by a comprehensive survey of local public health teams, capturing new data on current delivery models, including recent innovation in response to the Covid-19 pandemic;
- Our review may be limited by the richness and relevance of the evidence available in the literature;
- Survey response rates may be adversely affected by Covid-19 pressures and we will need to take steps to mitigate these wherever possible.

Background

Cardiovascular disease (CVD) causes a quarter of all deaths in the UK and is the largest cause of premature mortality in deprived areas. Early detection and prevention is an important priority for the NHS in England, and the NHS Long Term Plan (2019) commits to taking wider action on prevention to tackle the underlying causal factors. Over the course of 2020, it has also become clear that many risk factors associated with CVD, and existing health inequalities, are associated with poorer outcomes for Covid-19 patients.(1) The UK government have therefore highlighted the potential role that the NHS Health Check may have to play in helping to address these risk factors.(2)

The NHS Health Check programme is one of the main pillars of CVD prevention efforts in England. It was first launched in 2009, aiming to offer a 5-yearly assessment of individual risk of developing coronary heart disease, stroke, diabetes and chronic renal disease to the population aged between 40 and 74.(3) The Health Check involves measurement of key risk factors and calculation of CVD risk, followed by discussion and agreement on lifestyle and pharmacological approaches to managing the risk. This is a national, mandated programme, originally commissioned by Primary Care Trusts across England. The NHS Health Check programme was re-launched in 2013, when commissioning moved from Primary Care Trusts to Local Authorities (LAs), with an implementation review and action plan.(4) Although tests and measurements are standardised to help ensure the safety, quality and effectiveness of the programme,(5, 6) LAs have flexibility in how and who they commission to provide NHS Health Checks. As commissioning and delivery are determined locally, with the aim of meeting the needs of local populations, there is inevitable variation in delivery, uptake and outcomes.

The total eligible population for the Health Check programme has been estimated to be c. 15.5 million.(7) The largest and most recently published analysis of national data relating to the programme found that almost 10 million eligible people were offered an NHS Heath Check between 2012 and 2017.(8) Of these, 52.6% (just over 5 million) took up the offer. Although national uptake rates generally increased over this period, there was significant regional variation, with uptake rates calculated for upper tier LA areas ranging from 25.1% to 84.7%.(8) These findings are in line with previous analyses that have identified significant variation in invitation and uptake rate for the programme,(9) and variation in Health Check delivery and follow-up, including referrals to lifestyle

For peer review only

services.(10, 11) At present, minimal national data exist to explain these differences, yet it is important to determine what does work, for whom in what setting.

At the time of writing, Public Health England is undertaking a review of the NHS Health Checks, with the aim of making recommendations to improve both the content and delivery of the programme.(12, 13) The review will encompass a wide remit and include consideration of: whether additional 'checks' should be incorporated into the current offering; options to tailor the Health Check and personalise the programme for individuals; and digital tools that may offer opportunities to improve delivery. This review is being undertaken in a wider context of considerable uncertainty for national-level public health functions, following the announcement by Government in August 2020 that Public Health England will be dismantled and a new national institute for health protection created.(14, 15)

Overview of existing evidence

In 2014, Public Health England (PHE) established an Expert Scientific and Clinical Advisory Panel (ESCAP) to continually review the evidence on the NHS Health Check programme.(16) This group recommended periodic syntheses of published evidence. The first such review was a rapid evidence synthesis, published in 2017(11) and updated in 2020.(10) These reviews addressed six research questions identified by PHE, focused on uptake (questions 1-3), management of those at high risk (question 4), patient experience (question 5) and a specific set of outcomes related to the Health Check, including disease detection, referral, reductions in CVD risk and prescribing (question 6). The authors of both reviews identified significant gaps in the literature, and both ESCAP(17) and the review authors made recommendations for action and further research. These recommendations included:

i. Improved characterisation of local variations in implementation of the Health Check, to allow comparisons and sharing of best practice;(11)
ii. The need to develop a model that fully reflects the real-life NHS Health Check intervention and draws on current evidence to estimate its impact;(10, 17)
iii. The need for more research to determine the effect of the Health Check on lifestyle behaviour in different groups.(17)

These rapid evidence syntheses included documents identified by PHE using a systematic and comprehensive search strategy, updated each quarter.(18) Searching was conducted in multiple databases: MEDLINE, PubMed, Embase, HMIC, CINAHL, Global Health, PsycINFO, the Cochrane Library, NHS Evidence, Google Scholar, Google, Clinicaltrials.gov and the ISRCTN registry. The rapid reviews augmented these with additional searches in the Web of Science (Science Citation Index),(10, 11), OpenGrey(11) and a review of abstracts submitted to the 2017 PHE NHS Health Check conference. Together, the two reviews include evidence covering the period January 1996 to December 2019.

In addition to these reviews (and their associated academic publications,(9, 19-21), a further 3 reviews relating to the NHS Health Check have been published to date,(22-24) and we have also identified two other systematic reviews focused on participation and patient experience in similar prevention programmes.(25, 26) Drawing on the existing evidence, below we summarise what is currently known about NHS Health Check programme:

- **Coverage (proportion eligible who receive a Health Check)** is known to vary substantially across regions and settings, but is consistently higher in older people, females and in more deprived populations, although this may reflect targeting.(11) Studies suggest that

For peer review only

community outreach services can reach particular socio-demographic groups,(27) but one study suggests that these services may create inaccuracies in reporting.(28)

- **Uptake (attendance following invitation)** varies across regions and at GP practice level.(24) The evidence on uptake in different groups is highly heterogeneous. There is relatively consistent evidence that older people and women are more likely to take up invitations, but mixed findings in relation to ethnicity and deprivation, with some studies showing higher uptake in specific groups, while others show no difference.(10, 11, 24) There is also clear evidence that uptake is lower amongst smokers.(11, 24).
- **Invitations** are issued in different formats, though letters are most common.(29, 30) Recent studies of the effectiveness of different formats have found that modifications to standard letters, text message invitations/reminders, telephone and opportunistic face-to-face invitations can increase uptake.(10, 11, 29, 31) One cross-sectional study suggests that different invitation methods may be more or less effective for different ethnic and gender groups.(29) Telephone calls including the option to book an appointment during the call may overcome anticipated difficulties in making appointments and offer an opportunity to increase participants' understanding of the Health Check,(32) which may be barriers to uptake.(23) Other barriers may include aversion to preventive medicine, competing priorities and, for community pharmacy and outreach settings, concerns about privacy and confidentiality.(10, 11, 19, 23). Some qualitative evidence highlights the convenience of these settings and the value of community ambassadors.(10, 20)
- **Delivery** of the Health Check varies considerably across settings, despite the standardisation provided by PHE's guidance and the legislation that mandates its delivery. Providers delivering NHS Health Checks have reported challenges with workload, IT, funding, training and the need to cover multiple aspects within one consultation.(10, 11, 23, 33) A recent review found that although many providers recognised the importance of behaviour change to reduce CVD risk, professionals have different views on the contributions of behavioural versus pharmacological interventions, and on the clinical and cost-effectiveness of the Health Check programme.(23) Providers recognise the difficulty patients face in making sustained behaviour and lifestyle changes, acknowledging the need to take patients' social circumstances and resources into account.(34, 35) Professionals have also expressed concerns about limited access to appropriate lifestyle services for onward referrals, and about stretched resources and workload in primary care.(10, 11)
- **Patient experiences** are reported to be positive overall, with patient surveys indicating high levels of 'patient satisfaction' (consistently over 80%) and some reporting that attendance had precipitated lifestyle changes.(10, 11) However, qualitative studies have found that some patients report unmet expectations and confusion around follow up and risk scores. Some patients have found lifestyle advice too simplistic and un-personalised.(10, 11)
- **Outcomes** demonstrating clinical and/or cost effectiveness of the NHS Health Check are harder to obtain. Existing research demonstrates that the Health Check increases the detection of CVD risk factors and disease, and leads to increased statin prescribing (by 3-4%).(10, 11) Some studies also report increased prescribing of antihypertensive drugs (but one cohort study reported that Health Check attendees were *less* likely to receive antihypertensives than matched controls (36)). Three national studies found that the Health Check increased referrals to smoking cessation, weight management, exercise or alcohol support services.(36, 37) However, regional studies demonstrate wide variation in service availability and referral practice across England.(10, 11) The PHE-commissioned rapid reviews identified six primary studies that examined behaviour change, but smoking is the only health behaviour assessed. A limited number of studies have demonstrated post-Health

Check improvements in relevant risk factors, including BMI, diastolic blood pressure, total cholesterol and overall CVD risk, but results across studies are inconsistent and some have found no evidence of any effect.(10)

It is clear across the existing reviews that the emphasis in the literature is on the early steps of the Health Checks pathway, and especially on the invitation to, and uptake of the Health Check. This focus may reflect the variation that is apparent in the published indicators on Health Checks,(7) and emphasis on improving uptake in the legislation that mandates the programme.(38) Studies of Health Check delivery focus on patient and provider experience and perceptions, and there is more limited evidence on what happens after a Health Check. There is a notable absence of studies of post-Health Check behaviour change beyond smoking cessation. At all stages, the existing research demonstrates wide variation in implementation and practice, and significant uncertainties in relation to understanding this variation and the optimal strategies for increasing coverage and uptake, delivery models and maximising important patient outcomes.

Evidence explaining why this research is needed now

Since publication of the rapid evidence review in February 2017, and the rapid review update in 2020, several new studies have been published which add to the findings of the review. A PubMed search conducted in November 2020 for studies published since December 2019 has identified a further 8 empirical studies concerning the NHS Health Check(8, 39-45) and one protocol for an implementation study and trial.(46)

To improve our understanding of how the NHS Health Check achieves its outcomes, it is essential to learn as much as possible from how the programme is delivered, in different settings and by different providers. In the past, case studies have been used by PHE to illustrate 'good practice'.(4) To date, case studies shared by PHE on the Health Checks website (n=24 in November 2020) have focused on sharing practice in relation to increasing coverage or targeting Health Checks invitations to particular groups.(47) Selected local evaluations submitted to PHE have also been shared on the website (n=26 in November 2020), (48) and learning from local implementation of the Health Checks programme has regularly been shared at PHE-run conferences focused on the Health Check and CVD prevention.(49) In addition, a survey of commissioners and providers focused on targeting Health Checks was recently carried out by PHE(50), and a further survey seeking data on local delivery models is currently underway. Both add further local learning which could be used to help understand what works, for whom, how, and in what setting.

None of the reviews conducted to date have effectively utilised this abundant learning from the local level. The first rapid review looked at abstracts submitted to the 2017 NHS Health Check conference, but these form a minor aspect of the review.(11) More recently PHE commissioned the UCL Centre for Behaviour Change to conduct a review of barriers and facilitators to behaviours relevant to NHS Health Checks, including those of providers and invitees/attenders; again, this review included only papers published in academic journals.(23)

It is now eleven years since the Health Checks programme was launched. The amassed evidence from small, local unpublished studies needs to be combined with the published papers, many of which also cover only one locality. This evidence also needs to be combined with more comprehensive knowledge of the variety of ways in which different localities implement the NHS Health Check programme. It is by combining and analysing this evidence that we seek to answer the important research questions set out below.

For peer review only

Aim and objectives

The NHS Health Check programme is arguably one of the largest prevention programmes of its type in the world, and a cornerstone for the NHS prevention programme. However, many unanswered questions remain. To ensure that our research is sufficiently focussed and will produce findings that are relevant to knowledge users, we sought advice and feedback from colleagues at PHE and our wider stakeholder group to arrive at our aims, objectives and research questions.

Aim

To understand how the NHS Health Check programme works in different settings, for different groups, in order to recommend improvements to maximise intended outcomes.

Objectives

1. To conduct a realist review to enable understanding of how the NHS Health Check programme works in different settings, for different groups to achieve its outcomes.
2. To map how the programme is currently delivered across England, using data collected in a PHE survey (in October 2020), and data we will collect using our own online survey of Local Authorities.
3. To provide recommendations on tailoring, implementation and design strategies to improve the current delivery and outcomes of the NHS Health Check programme in different settings, for different groups.

Review Questions

1. What are the mechanisms by which the current NHS Health Check programme produces its intended outcomes?
2. What are the important contexts which determine whether the different mechanisms produce intended outcomes?
3. In what circumstances are such interventions likely to be effective?

Methods and analysis

Objective 1: To conduct a realist review

The plan of investigation will follow this protocol which is informed by Pawson's five iterative stages in realist reviews (see Figure 1 below).(51) We have chosen to use a realist review approach because the existing research indicates that the NHS Health Check programme is a complex intervention that has a range of outcomes (e.g. variable rates of attendance, follow-up, onwards referral, prescription) which are context sensitive and vary for different groups. We are also aware that the NHS Health Check programme is continually evolving: work exploring the potential for digital services has been under consideration since 2017,(52) and a wide-ranging review of the Health Check programme is currently underway and expected to make recommendations in early 2021.(12) In addition to this, it is clear that the pause and restart of the Health Check programme during the Covid-19 pandemic has provoked a range of responses at local levels, including the introduction of new delivery models in some areas.(53)

Any evidence synthesis that seeks to make sense of how to improve the tailoring, implementation and design of the NHS Health Check programme must take into account the various contexts and interventional strategies in which NHS Health Checks are delivered and the varied outcomes for

For peer review only

different groups. A realist review will be able to generate the knowledge needed to address both these issues.(54) Realist review is an interpretive, theory-driven approach to synthesizing evidence from qualitative, quantitative and mixed-methods research. Its main strength comes from providing findings that coherently and transferably explain how and why context can influence outcomes.

This process of explanation building starts with the development and refinement of a realist ‘programme theory’ of the NHS Health Check programme. To do this, we have ‘mapped’ the sequence of steps needed to achieve the final intended outcomes for the programme, taking account of the processes outlined by PHE in their implementation guidance (see Figure 2 below). This initial programme theory will be refined (see Step 1 below) and then further refined and tested against empirical evidence during the review (see Steps 2-5).

Patient and public involvement

Throughout the review, we will consult with a wide range of stakeholders with content expertise and a variety of perspectives. We will invite policy makers, commissioners, training providers, and front-line providers of the Health Checks programme, as well as representatives from relevant charities and members of the public drawn from the eligible population. We will update and extend the membership of this group as needed over the course of the review. We have secured the participation of PHE as a key stakeholder and they have expressed strong interest in assisting us during the review and in our findings. Our initial programme theory will be presented to our stakeholder group and refined based on their feedback. As the programme theory is further refined over the course of the review, the stakeholders will be regularly consulted on the developing findings. The group will meet (either virtually or face to face) four times during the study and communicate via e-mail as necessary. The meetings will be chaired by GW or VH (our PPI Lead, a member from the Public Involvement in Pharmacy Studies at Medway School of Pharmacy group).

This review protocol has been registered with PROSPERO (registration number CRD42020163822) and will follow current quality and publication standards.(55) An overview of the project may be found in **Figure 1** below, and the main steps involved in the review are outlined in more detail below.

[Figure 1]

Step 1: Locate existing theories

The goal of this step is to identify theories that explain how the NHS Health Check programme is supposed to work (and for whom), when it does work, when it does not achieve desired changes in clinical practice and patient outcomes, why it is not effective, and why it is not being used.(56) The rationale for this step is that interventions are “theories incarnate” – that is underpinning the design of such interventions are theories of why certain components are required. In other words, the designers of the programme have put it together in a certain way based on their theories about what needs to be done to get one or more desired outcomes.(57) For example, one theoretical assumption underpinning the programme design is that once patients are given information about their cardiovascular risk score, this knowledge will motivate a proportion of them to make the necessary lifestyle changes to reduce it.(58) There are a number of different theories and models that support such an assumption, though evidence highlights the complexity of achieving sustained behaviour change (e.g.(59)).

To locate relevant theories, we will iteratively:

a) consult with key content experts in our stakeholder group, and

For peer review only - http://bmjopen.bmj.com/site/about/guidelines.xhtml

b) informally search the literature to identify existing theories, including both grey literature in the form of NHS Health Check programme documentation, and published research that has employed formal or substantive theory to understand the programme.

This informal searching differs from the more formal searching process described below in Step 2; it is exploratory and aims to quickly identify a range of possible explanatory theories that may be relevant. Search methods such as citation tracking and snowballing,(60) along with more structured searching for theories(61, 62) will be used.

From these, we will refine an initial programme theory to test in the review. A first version of this initial programme theory is outlined as a starting point in **Figure 2** below. In Step 1, we will refine this model within the project team, and present it to our stakeholder group for their feedback.

[Figure 2]

Focus of the review

To develop this protocol, we undertook searches to identify existing reviews related to the Health Checks programme. In so doing, we identified an existing focus on the early steps in our initial programme theory, and especially on *invitation* and *uptake* of the NHS Health Check (see Figure 2). Conversely, there has been less focus on what happens next, in relation to follow up, onward referral and ongoing support for lifestyle and behaviour change. As a result, we have decided to focus our review efforts on these later steps, which are crucial in delivering the programme's intended outcomes, in relation to supporting individuals to make changes to their behaviour and lifestyle, and ultimately to reduce their risk of experiencing a heart attack, stroke or developing some forms of dementia.(6) We have confirmed the value of this focus with PHE, our key stakeholder, and we will seek further feedback and advice from our wider stakeholder group to help us to develop our thinking about this stage of the Health Checks pathway. However, in recognition of the complex nature of the Health Checks programme, and the possibility that feedback loops may exist, such that the characteristics of one step may be inextricably bound up with others, we will not exclude evidence relating to any other programme steps, but will actively seek out data that might shed light on relationships between each step.

As noted above, we are also aware that the Health Checks programme is continually evolving, and especially aware of the effects of the Covid-19 pandemic on programme delivery, and that a major review of the programme began in August 2020. PHE and the Department of Health and Social Care have acknowledged that many of the factors assessed by the NHS Health Check are also risk factors for severe Covid-19, and the fact that the programme has an explicit aim to reduce health inequalities(2, 13), now a pressing agenda. In addition, restrictions on face-to-face contact, and increased pressure on health services have created a new impetus to adapt programme delivery and may speed up the proposed shift to introduce digital methods or other innovations. Our own review project is taking place within this fast-moving context, and as such, we will aim to prioritise the inclusion of evidence that can support findings and recommendations with relevance for the 'new normal'.

Step 2: Search for evidence

Formal search

The purpose of this Step is to find a relevant 'body of literature' with which to further develop and refine the initial programme theory from Step 1. The search strategy used will be designed, piloted and conducted by CD, in collaboration with the project team. CD is an information specialist with

For peer review only

extensive experience of conducting searches for complex systematic reviews, particularly realist reviews.

At the outset of this project, we were aware that Public Health England regularly undertakes literature searching for new evidence relating to the NHS Health Check. These regular searches employ a very sensitive search strategy across 13 relevant sources (PubMed, MEDLINE, Embase, HMIC, CINAHL, Global Health, PsycINFO, Cochrane Library, NHS Evidence, Google Scholar, Google, Clinicaltrials.gov and the ISRCTN registry).(18) These searches have been used in previous review projects commissioned by PHE to identify evidence relating to the NHS Health Check.(10, 11) These reviews have included additional searches in OpenGrey and/or Web of Science (Science Citation Index); the most recent review captured studies published until the end of December 2019.(10)

We do not intend to duplicate this work, but instead aim to re-use and extend it as follows:

1. We will re-use existing searches by considering for inclusion all documents included in the two existing reviews commissioned by PHE (n=98 documents). These were empirical (quantitative and qualitative) studies of the NHS Health Check. The eligibility criteria employed in each of these reviews is summarised below in **Table 1**.

Table 1: Summary of eligibility criteria for commissioned rapid reviews of the NHS Health Check

Inclusion criteria	Exclusion criteria
Studies of the NHS Health Check<br>Populations including those eligible for, attending, not attending and providing NHS Health Checks<br>Study designs:<br>RCTs, quasi-RCTs<br>Before and after studies with appropriate comparator groups<br>Interrupted time series<br>Cohort studies (prospective or retrospective)<br>Case-control studies<br>Qualitative studies using recognised methods<br>Economic and health outcome modelling	Editorials, commentaries and opinion pieces

2. We will then seek additional material to consider for inclusion:
   a. We will run additional targeted searches focused on identifying material related to the NHS Health Check that was excluded from the existing reviews. This may include, for example: relevant commentary or opinion, which are not excluded from realist reviews as they may contribute to theory building; studies focused on aspects of the Health Check not covered by the existing reviews; and studies published since December 2019. We will use specific searches to identify additional documents focused on the Health Check programme in England, using specific free text terms describing the Health Check programme alongside relevant subject heading terms as appropriate. We will search MEDLINE, Embase, CINAHL, HMIC and Web of Science (Science and Social Science Citation Indexes). The full details of the search strategies for these searches are available in the online **Supplementary File**.
   b. We will trawl the NHS Health Checks website for documents including case studies (n=24 in November 2020), local evaluations (n=24 in November 2020) and abstracts and posters presented at the Health Checks/Cardiovascular Disease Prevention annual

For peer review only - http://bmjopen.bmj.com/site/about/guidelines.xhtml

conferences from 2014-2020 (n>450), where these are accessible via current and archived versions of the website. These sources represent an important source of data on local implementations of the Health Check that has been excluded from previous reviews.

c. For each included published document, we will undertake backwards and forwards citation searches, i.e. for these documents, we will screen their reference lists, and use Google Scholar's 'cited by' links to identify future documents that cited these. Where necessary, we will also undertake further searching to identify 'sibling' documents, related to those that we have already included by virtue of being part of the same broader research projects.(62)

3. If necessary (i.e. if further data is required for programme theory development) we will continue to seek additional material as follows:
   a. We will consider for inclusion all additional studies focused on Health Check (and related international programmes and evidence) included in PHE's published quarterly literature reviews from October 2014 onwards available on the Health Checks website (including those published during our own review project, to ensure the most up to date material was included).
   b. We will run more targeted searches for additional grey literature, including, for example, searching additional relevant websites (Department of Health and Social Care, NHS England, CCGs and LAs).
   c. We will seek access to unpublished evaluation reports by contacting LA public health teams directly (see Objective 2 below for more details).

To ensure we keep up to date with emerging material as the review progresses, we will set up a regular search alert (via Google Scholar) and continue to consult PHE's regularly published literature reviews (via the Health Checks website).

Screening

For the material and searches described above, our inclusion and exclusion criteria are broad as we seek to find quantitative, qualitative and mixed-methods documents, and relevant grey literature. The following criteria will be applied:

Inclusion:

- Intervention: NHS Health Check programme (all delivery models, including face to face and digital)
- Study design: all study designs
- Setting: any setting providing NHS Health Checks in England
- Participants: all adults eligible for NHS Health Checks
- Outcome measures: all outcome measures related to NHS Health Checks

Exclusion:

- Cardiovascular screening programmes run in countries other than England
- Other NHS screening programmes
- Routine health checks offered to specific target populations by the NHS which are not part of the NHS Health Check programme

For peer review only

Screening will be undertaken by CD, based on title and abstract, with a 10% random sample of the citations retrieved from searching will be reviewed independently for quality control by GW. Where necessary (e.g. no abstract is available) the full text of a document will be consulted. Any disagreements about inclusion will be resolved by discussion. If disagreements remain, the matter will be presented to JK and resolved by majority vote.

Additional searching

An important process in realist reviews is searching for additional data to inform programme theory development. In other words, more searches may be undertaken if we find that we require more data to develop and test certain parts of our programme theory. Some of our proposed strategies for identifying additional data are listed above (point 3), but this list is not necessarily exhaustive. As our programme theory will take into account the wider contextual factors that impact on outcomes from the NHS Health Check, we may also run searches to identify additional data on relevant contexts. For example, we anticipate that we may need to seek evidence relating to the commissioning process, relationships between Health Checks providers (including GPs) and LAs, interprofessional relationships, and the presence of other prevention programmes and related services, which may interact, or overlap with the Health Check.

This additional searching will greatly increase the amount of relevant data available to us for the realist review. For any additional searching undertaken, the project team will discuss and set inclusion and exclusion criteria. CD will develop, pilot and refine additional search strategies as needed. Screening will be conducted as described above. As in Step 1, these searches are likely to be exploratory and purposive, potentially seeking documents from a wide range of disciplines. Where applicable, we will follow search strategies described by Booth et al, developed for just such data.(62)

Step 3: Article selection

Following initial screening, documents will be read in full text and selected for inclusion in the review based on an assessment of *relevance* (whether data can contribute to theory building and/or testing) and *rigour* (whether the methods used to generate the relevant data are credible and trustworthy).(63) Even when a document found from the initial search has been screened and has met the inclusion criteria, it may still not contain any data that is relevant for programme theory development and refinement.

CD will read the full text of all the documents that have been included after initial screening. Documents will be selected for inclusion when they contain data that is relevant to the realist analysis – i.e. could inform some aspect of the programme theory. At the point of inclusion based on relevance, an assessment will also be made of rigour (how trustworthy were methods used to generate the data). To illustrate how we will operationalise the assessment of rigour: if data have been generated using a questionnaire/survey, the trustworthiness of the data will be considered to be higher if the questionnaire had previously been shown to be reliable and valid, and remained unaltered (or where subsequent testing had been undertaken following any alterations). However, data may still be included even if judged to be of limited rigour, as we will also make an overall assessment of rigour at the level of the programme theory.(64) In other words, we will also consider the role that each piece of data plays in our developing programme theory and how it strengthens (or not) our explanations of outcomes.

A random sample of 10% of documents identified as including relevant data will be selected, assessed and discussed between CD and GW to ensure that decisions to finally include have been

For peer review only

made consistently. The remaining 90% of decisions will be made by CD (though a number of these may require further discussion and joint reading within the project team, where there is any uncertainty over issues of relevance or rigour. As necessary, we will employ the same decision-making processes as were used during screening in Step 2.

Step 4: Extracting and organising data

The main characteristics (bibliographic details and information relating to study design, participants, settings and findings) of the included documents will be extracted into an Excel spreadsheet.

The full text of included documents will be uploaded into NVivo (a qualitative data analysis software tool). Relevant sections of text in these documents will be coded in NVivo, and interpreted as relating to contexts, mechanisms and outcomes, or relationships between these. Coding will be both deductive (codes created in advance of data extraction and analysis will be informed by the initial programme theory), inductive (codes will be created to categorise data reported in included studies), and retroductive (codes will be created based on an interpretation of data, to infer the causal forces that generate observed outcomes, i.e. mechanisms). Each new element of data will be used to refine the theory if appropriate, and as the theory is refined, included studies will be re-scrutinised to search for data relevant to the revised theory that may have been missed.

Data extraction and organisation will be undertaken by CD. As with screening and inclusion decisions, a random sample of 10% of documents will be independently checked by GW for quality control. Any disagreements will be resolved by discussion and if disagreements remain JK will be asked for her opinion, and resolution will be by majority vote.

Step 5: Synthesising the evidence and drawing conclusions

We will use a realist logic of analysis to make sense of data included in the review. CD will undertake this step with support from GW and EG. We will use a series of questions about the relevance and rigour of content within data sources as part of our process of analysis and synthesis(65):

- Relevance: Are sections of text within this document relevant to programme theory development?
- Rigour (judgements about trustworthiness): Are these data sufficiently trustworthy to warrant making changes to any aspect of the programme theory?
- Interpretation of meaning: If the section of text is relevant and trustworthy enough, do its contents provide data that may be interpreted as functioning as context, mechanism or outcome?
- Interpretations and judgements about Context-Mechanism-Outcome-Configurations (CMOCs): What is the CMOC (partial or complete) for the data that has been interpreted as functioning as context, mechanism or outcome? Are there further data to inform the particular CMOCs contained within this document or other documents? If so, which other documents? How does this particular CMOC relate to other CMOCs that have already been developed?
- Interpretations and judgements about programme theory: How does this particular (full or partial) CMOC relate to the programme theory? Within this same document are there data which inform how the CMOC relates to the programme theory? If not, are there data in other documents? Which ones? In light of this particular CMOC and any supporting data, does the programme theory need to be changed?

For peer review only

Data to inform our interpretation of the relationships between contexts, mechanisms and outcomes will be sought not just within the same document, but across documents (e.g. mechanisms inferred from one document could help explain the way contexts influenced outcomes in a different document). Synthesising data from different documents is often necessary to compile CMOCs, since not all parts of the configurations will always be articulated in the same document.

Within the analytic process set out above, we will use interpretive cross-case comparison to understand and explain how and why reported outcomes have occurred, for example, by comparing the elements within the NHS Health Check programme which have produced a particular outcome against those which have not, to understand how context has influenced reported findings. When working through the questions set out, where appropriate we will use the following forms of reasoning to make sense of the data:

- Juxtaposition of data: for example, where data about how Health Checks setting influenced outcome in one document enables insights into data about outcomes in another document.
- Reconciling of data: where data differ in apparently similar circumstances, further investigation is appropriate in order to find explanations for why these differences have occurred.
- Adjudication of data: where there are conflicting data, plausibility of these data can be informed on the basis of methodological strengths or weaknesses of the data collection methods.
- Consolidation of data: where outcomes differ in particular contexts, an explanation can be constructed of how and why these outcomes occur differently.

In addition to the material identified for inclusion in the review, additional information obtained via a survey will also provide rich data on contexts and mechanisms in different localities, which will enhance our ability to make sense of the data from the documents identified in the searches. See Objective 2 below for more details.

Objective 2: To map current delivery across England

This objective will both enable additional material (local knowledge, unpublished evaluations and examples of best practice and Covid-related innovation) to be identified for the review and provide a comprehensive overview of how different localities across England implement the NHS Health Check programme. It will be conducted (with support from the project team and stakeholder group) by EG, a senior public health researcher with a strong understanding of LA commissioning and public health service provision.

PHE conducted a survey of LA commissioners in October 2020 as part of the wider review of the Health Checks programme. From this survey, they have provided detailed information from the 104 responding authorities related to some aspects of the programme's delivery from April 2019 until March 2020, in particular: how the eligible population was identified; what methods were used for first invitation; when and in which settings Health Check appointments were made available, and who provided them; whether and what type of point of care testing was used; whether digital solutions were used in delivery; and how the provider workforce was supported. PHE and their survey respondents have granted us permission to include these data in our study.

We will supplement this existing information with our own survey, by asking questions related to current delivery models (in 2021, following the Covid-19 related pause to the service) and questions related to options for onward referral and follow-up of patients after the Health Check encounter. In particular, our survey will identify the extent to which commissioners and providers are changing the

For peer review only

way they commission and deliver the NHS Health Check programme in light of the Covid-19 pandemic. We will also identify the extent to which services are available to support those identified as having modifiable risk factors, which will help us to address our review focus on what happens after a Health Check, especially in relation to follow up, onward referral and ongoing support for lifestyle and behaviour change.

Our survey will be designed in collaboration with our stakeholders and pilot tested prior to being distributed as an online survey (using Jisc Online Surveys) to all 152 upper-tier and unitary LAs in England. To ensure a maximal response, and to ensure the survey is correctly targeted to those who can answer it, we will work closely with PHE to distribute the survey and make use of their tried-and-tested processes for dissemination. PHE have agreed to send the survey on our behalf through their local networks and send up to two reminders to non-responders. They will also publicise it through their established national and local communications channels (including the NHS Health Check e-Bulletin, website and Twitter feed).

Survey responses will be logged, managed and sorted for analysis using Microsoft Excel. Where there are missing data from completed surveys, we will search each LA's website to see if we can find the necessary information. Resources and time permitting, we will also search for data from the websites of LAs which have not responded to the survey. The information gained from our survey will be combined with the data from PHE's earlier survey and analysed in SPSS. This will enable us to understand the different ways in which the Health Checks programme is delivered in different contexts and to develop a comprehensive picture of how the policy intent of the programme is translated into practice across England. We anticipate mapping provision against uptake and other elements of the national dataset, which will be available at LA level.

As part of our survey, we will request copies of local evaluations to add to the literature obtained through searching for the realist review. If these local evaluations fulfil our inclusion criteria they will be included in the review (see Step 2 of Objective 1 above for more details).

Objective 3: To provide recommendations

Our programme theory will be used to develop recommendations on tailoring, implementation and design strategies to improve the current delivery and outcomes of the NHS Health Checks programme in different settings, for different groups. Further details are provided in the Ethics and Dissemination section below.

Ethics and dissemination

Dissemination

Our dissemination strategy will build on the participatory approach we have adopted throughout the review process, involving the stakeholders that have engaged with us during the development of this research proposal and throughout the review process. Our approach will be integrative, valuing the different forms of knowledge that are required to produce findings capable of informing complex decision making.(66) A range of audiences will be interested in the review's findings and recommendations, including:

a. Policy makers, decision-makers and commissioners of NHS Health Checks;
b. Providers of the NHS Health Check and related lifestyle services;
c. Members of the public, including those eligible for NHS Health Checks and relevant advocacy organisations

For peer review only

Different strategies are likely to be needed for each of these. This project will produce three major types of outputs in addition to the final report:

a. Academic outputs;
b. A range of audience-specific ‘How to’ publications that outline practical advice on tailoring, implementation and design strategies to enhance current NHS Health Check delivery;
c. User-friendly summaries of the review findings tailored to the needs of the different audiences.

We will draw on the advice and expertise within our stakeholder group to help clarify the main ‘players’ for dissemination for each audience, and to develop tailored and relevant materials for each group.

Ethics

Ethical approval for the survey component of our study has been granted by the University of Kent’s SRC Research Ethics Committee (SRCEA id 0367).

For peer review only

Author contributions

GW and JK conceived the study with input from EG and CD. GW and JK led the design and wrote the first draft of this protocol and JK, EG, CD and VH provided criticism and refinement. CD designed the search strategy. All the authors have read and approved the final manuscript.

Funding statement

This project is funded by the National Institute for Health Research (NIHR) Health Services and Delivery Research Programme (Grant Reference Number NIHR 129209). The views expressed in this publication are those of the author(s) and not necessarily those of the NIHR or the Department of Health and Social Care.

Competing interests

CD and GW are both members of the Royal College of General Practitioners (United Kingdom) Overdiagnosis and Overtreatment Group.

GW is an NHS General Practitioner and deputy chair of the National Institute for Health Research Health Technology Assessment Prioritisation Committee: Integrated Community Health and Social Care Panel (A).

Figure legends

Figure 1: Project flow diagram

Figure 2: Initial programme theory behind NHS Health Checks and some of the processes that may influence outcomes

For peer review only

References

1. NHS. Who's at higher risk from coronavirus 2020 [Available from: https://www.nhs.uk/conditions/coronavirus-covid-19/people-at-higher-risk/whos-at-higher-risk-from-coronavirus/.
2. Cabinet Office. Our plan to rebuild: The UK Government's COVID-19 recovery strategy. 2020.
3. NHS. NHS Health Check 2020 [Available from: https://www.nhs.uk/conditions/nhs-health-check/.
4. Public Health England. NHS Health Check implementation review and action plan Summary. 2013.
5. Public Health England. NHS Health Check programme standards: a framework for quality improvement July 2020. 2020.
6. Public Health England. NHS Health Check Best practice guidance for commissioners and providers October 2019. 2019.
7. Public Health England. NHS Health Checks Fingertips Profile 2020 [Available from: https://fingertips.phe.org.uk/profile/nhs-health-check-detailed.
8. Patel R, Barnard S, Thompson K, Lagord C, Clegg E, Worrall R, et al. Evaluation of the uptake and delivery of the NHS Health Check programme in England, using primary care data from 9.5 million people: a cross-sectional study. BMJ Open. 2020;10(11):e042963.
9. Martin A, Saunders CL, Harte E, Griffin SJ, MacLure C, Mant J, et al. Delivery and impact of the NHS Health Check in the first 8 years: a systematic review. Br J Gen Pract. 2018;68(672):e449-e59.
10. Tanner L, Kenny R, Still M, Pearson F, Bhardwaj-Gosling R. NHS Health Check Programme Rapid Review Update. University of Sunderland, Newcastle University, Public Health England; 2020.
11. Usher-Smith JA, Mant J, Martin A, Harte E, MacLure C, Meads C, et al. NHS Health Check Programme rapid evidence synthesis. Cambridge, UK: The Primary Care Unit, University of Cambridge, RAND Europe, Public Health England; 2017.
12. Cabinet Office, Department of Health and Social Care. Advancing our health: prevention in the 2020s - consultation document. 2019.
13. Public Health England. NHS Health Check e-Bulletin - August 2020 2020 [Available from: https://www.nhshealthcheck.nhs.uk/nhs-health-check-e-bulletin-august-2020/front-page/nhs-health-check-e-bulletin-august-2020.
14. Government creates new National Institute for Health Protection [press release]. 18 August 2020 2020.
15. Department of Health & Social Care. The future of public health: the National Institute for Health Protection and other public health functions. 2020 15 September 2020.
16. Public Health England. NHS Health Check: our approach to the evidence. 2013 July 2013.
17. Public Health England. Emerging evidence on the NHS Health Check: findings and recommendations. A report from the Expert Scientific and Clinical Advisory Panel. 2017.
18. Public Health England. NHS Health Check: Literature Review 2020 [Available from: https://www.healthcheck.nhs.uk/commissioners-and-providers/evidence/literature-review/.
19. Harte E, MacLure C, Martin A, Saunders CL, Meads C, Walter FM, et al. Reasons why people do not attend NHS Health Checks: a systematic review and qualitative synthesis. Br J Gen Pract. 2018;68(666):e28-e35.
20. Usher-Smith JA, Harte E, MacLure C, Martin A, Saunders CL, Meads C, et al. Patient experience of NHS health checks: a systematic review and qualitative synthesis. BMJ Open. 2017;7(8):e017169.
21. Mills K, Harte E, Martin A, MacLure C, Griffin SJ, Mant J, et al. Views of commissioners, managers and healthcare professionals on the NHS Health Check programme: a systematic review. BMJ Open. 2017;7(11):e018606.

For peer review only

22. Cooper A, Dugdill L. Evidence of improved uptake of health checks: rapid review. University of Salford,; 2014.
23. Atkins L, Stefanidou C, Chadborn T, Thompson K, Michie S, Lorencatto F. Influences on NHS Health Check behaviours: a systematic review. BMC Public Health. 2020;20(1):1359.
24. Bunten A, Porter L, Gold N, Bogle V. A systematic review of factors influencing NHS health check uptake: invitation methods, patient characteristics, and the impact of interventions. BMC Public Health. 2020;20(1):93.
25. Shaw RL, Holland C, Pattison HM, Cooke R. Patients' perceptions and experiences of cardiovascular disease and diabetes prevention programmes: A systematic review and framework synthesis using the Theoretical Domains Framework. Soc Sci Med. 2016;156:192-203.
26. de Waard AM, Wandell PE, Holzmann MJ, Korevaar JC, Hollander M, Gornitzki C, et al. Barriers and facilitators to participation in a health check for cardiometabolic diseases in primary care: A systematic review. Eur J Prev Cardiol. 2018;25(12):1326-40.
27. Woringer M, Cecil E, Watt H, Chang K, Hamid F, Khunti K, et al. Evaluation of community provision of a preventive cardiovascular programme - the National Health Service Health Check in reaching the under-served groups by primary care in England: cross sectional observational study. BMC Health Serv Res. 2017;17(1):405.
28. Riley VA, Gidlow C, Ellis NJ. Uptake of NHS health check: issues in monitoring. Prim Health Care Res Dev. 2018:1-4.
29. Cook EJ, Sharp C, Randhawa G, Guppy A, Gangotra R, Cox J. Who uses NHS health checks? Investigating the impact of ethnicity and gender and method of invitation on uptake of NHS health checks. Int J Equity Health. 2016;15:13.
30. Gidlow C, Ellis N, Randall J, Cowap L, Smith G, Iqbal Z, et al. Method of invitation and geographical proximity as predictors of NHS Health Check uptake. Journal of public health (Oxford, England). 2015;37(2):195-201.
31. Mason A, Liu D, Marks L, Davis H, Hunter D, Jehu LM, et al. Local authority commissioning of NHS Health Checks: A regression analysis of the first three years. Health Policy. 2018;122(9):1035-42.
32. Brangan E, Stone TJ, Chappell A, Harrison V, Horwood J. Patient experiences of telephone outreach to enhance uptake of NHS Health Checks in more deprived communities and minority ethnic groups: A qualitative interview study. Health Expect. 2019;22(3):364-72.
33. Chatterjee R, Chapman T, Brannan MG, Varney J. GPs' knowledge, use, and confidence in national physical activity and health guidelines and tools: a questionnaire-based survey of general practice in England. Br J Gen Pract. 2017;67(663):e668-e75.
34. Alageel S, Gulliford MC, McDermott L, Wright AJ. Implementing multiple health behaviour change interventions for cardiovascular risk reduction in primary care: a qualitative study. BMC Fam Pract. 2018;19(1):171.
35. Ismail H, Atkin K. The NHS Health Check programme: insights from a qualitative study of patients. Health Expectations. 2016;19(2):345-55.
36. Alageel S, Gulliford MC. Health checks and cardiovascular risk factor values over six years' follow-up: Matched cohort study using electronic health records in England. PLoS Med. 2019;16(7):e1002863.
37. Robson J, Dostal I, Sheikh A, Eldridge S, Madurasinghe V, Griffiths C, et al. The NHS Health Check in England: an evaluation of the first 4 years. BMJ Open. 2016;6(1):e008840.
38. The Local Authorities (Public Health Functions and Entry to Premises by Local Healthwatch Representatives) Regulations, Stat. 351 (2013).
39. Sallis A, Gold N, Agbebiyi A, James RJE, Berry D, Bonus A, et al. Increasing uptake of National Health Service Health Checks in primary care: a pragmatic randomized controlled trial of enhanced invitation letters in Northamptonshire, England. J Public Health (Oxf). 2019.
40. Alageel S, Gulliford MC, Wright A, Khoshaba B, Burgess C. Engagement with advice to reduce cardiovascular risk following a health check programme: A qualitative study. Health Expect. 2020;23(1):193-201.

For peer review only

41. Chattopadhyay K, Biswas M, Moore R. NHS Health Check and healthy lifestyle in Leicester, England: analysis of a survey dataset. Perspect Public Health. 2020;140(1):27-37.
42. Gidlow CJ, Ellis NJ, Cowap L, Riley VA, Crone D, Cottrell E, et al. Quantitative examination of video-recorded NHS Health Checks: comparison of the use of QRISK2 versus JBS3 cardiovascular risk calculators. BMJ Open. 2020;10(9):e037790.
43. Hardeman W, Mitchell J, Pears S, Van Emmenis M, Theil F, Gc VS, et al. Evaluation of a very brief pedometer-based physical activity intervention delivered in NHS Health Checks in England: The VBI randomised controlled trial. PLoS Med. 2020;17(3):e1003046.
44. Palladino R, Vamos EP, Chang KC, Khunti K, Majeed A, Millett C. Evaluation of the Diabetes Screening Component of a National Cardiovascular Risk Assessment Programme in England: a Retrospective Cohort Study. Sci Rep. 2020;10(1):1231.
45. Paxton B, Mills K, Usher-Smith JA. Fidelity of the delivery of NHS Health Checks in general practice: an observational study. BJGP Open. 2020;4(4).
46. Nahar P, van Marwijk H, Gibson L, Musinguzi G, Anthierens S, Ford E, et al. A protocol paper: community engagement interventions for cardiovascular disease prevention in socially disadvantaged populations in the UK: an implementation research study. Glob Health Res Policy. 2020;5:12.
47. Public Health England. NHS Health Checks - Case Studies 2020 [Available from: https://www.healthcheck.nhs.uk/commissioners-and-providers/evidence/case-studies/.
48. Public Health England. NHS Health Check - Local evaluations 2020 [Available from: https://www.healthcheck.nhs.uk/commissioners-and-providers/evidence/local-evaluation/.
49. Public Health England. NHS Health Checks - Webinars and Conferences 2020 [Available from: https://www.healthcheck.nhs.uk/commissioners-and-providers/events/.
50. England PH. NHS Health Check commissioning: Review of commissioner's current and potential use of weighted financial remuneration. 2018.
51. Pawson R, Greenhalgh T, Harvey G, Walshe K. Realist review - a new method of systematic review designed for complex policy interventions. Journal of Health Services Research & Policy. 2005;10(1_suppl):21-34.
52. Public Health England. NHS Health Checks - NHS Health Check Programme Digital Exemplar 2020 [Available from: https://www.healthcheck.nhs.uk/nhs-health-check-digital-exemplar/.
53. Public Health England. NHS Health Check: Restart Preparation [Webinar]. 2020.
54. Wong G. Is complexity just too complex? J Clin Epidemiol. 2013;66(11):1199-201.
55. Wong G, Greenhalgh T, Westhorp G, Buckingham J, Pawson R. RAMESES publication standards: realist syntheses. BMC Medicine. 2013;11(1):21.
56. Pawson R, Owen L, Wong G. The Today Programme's Contribution to Evidence-based Policy. Evaluation. 2010;16(2):211-3.
57. Pawson R. Realist Methodology: The Building Blocks of Evidence. 2006 2020/11/10. In: Evidence-based policy: A realist perspective [Internet]. London: SAGE Publications Ltd. Available from: https://methods.sagepub.com/book/evidence-based-policy.
58. Department of Health. Economic Modelling for Vascular Checks: A technical consultation on the work undertaken to establish the clinical and cost effectiveness evidence base for the Department of Health's policy of vascular checks. In: Health. Do, editor. 2008.
59. Michie S, Atkins L, West R. The behaviour change wheel: a guide to designing interventions. London: Silverback Publishing; 2014.
60. Greenhalgh T, Peacock R. Effectiveness and efficiency of search methods in systematic reviews of complex evidence: audit of primary sources. BMJ. 2005;331(7524):1064-5.
61. Booth A, Carroll C. Systematic searching for theory to inform systematic reviews: is it feasible? Is it desirable? Health Information & Libraries Journal. 2015;32(3):220-35.
62. Booth A, Harris J, Croot E, Springett J, Campbell F, Wilkins E. Towards a methodology for cluster searching to provide conceptual and contextual "richness" for systematic reviews of complex interventions: case study (CLUSTER). BMC Medical Research Methodology. 2013;13(1):118.

For peer review only

63. Pawson R. Realist synthesis: new protocols for systematic review. 2006. In: Evidence-based policy: a realist perspective [Internet]. [73-104].
64. Wong G. Data gathering in realist reviews: Looking for needles in haystacks. 2018. In: Doing Realist Research [Internet]. London: SAGE Publications Ltd; [131-46].
65. Papoutsi C, Mattick K, Pearson M, Brennan N, Briscoe S, Wong G. Interventions to improve antimicrobial prescribing of doctors in training (IMPACT): a realist review. NIHR Health Services and Delivery Research,. 2018.
66. Bowen S, Graham I. Integrated knowledge translation. 2013. In: Knowledge Translation in Health Care [Internet]. John Wiley & Sons; [14-23].

For peer review only

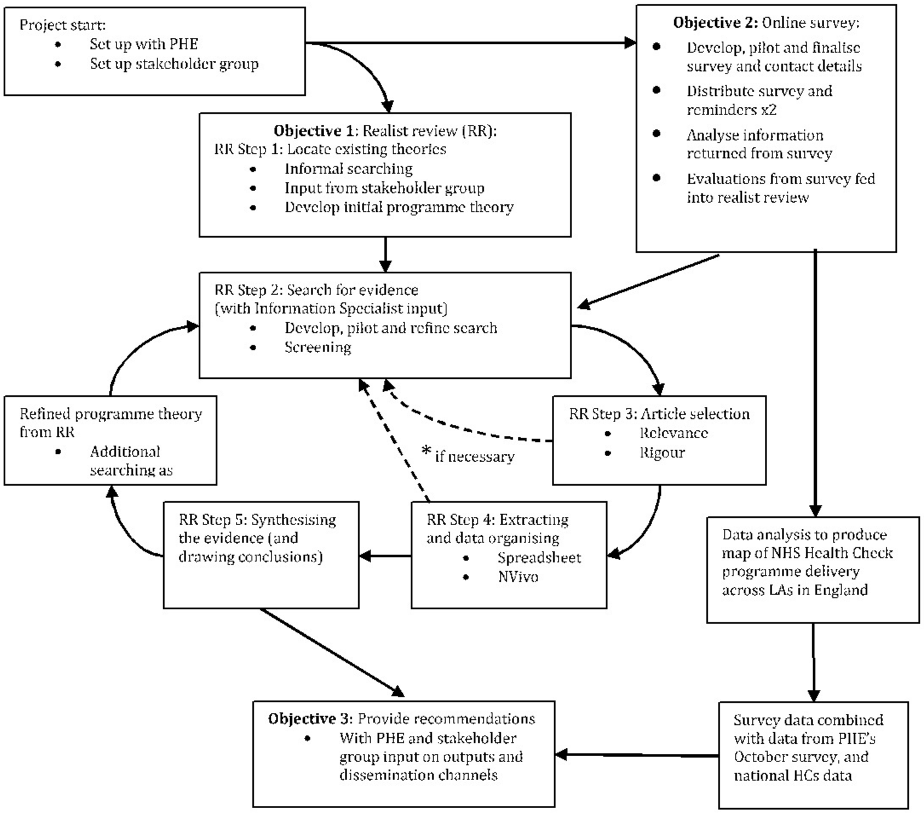

Figure 1: Project flow diagram

248x220mm (120 x 120 DPI)

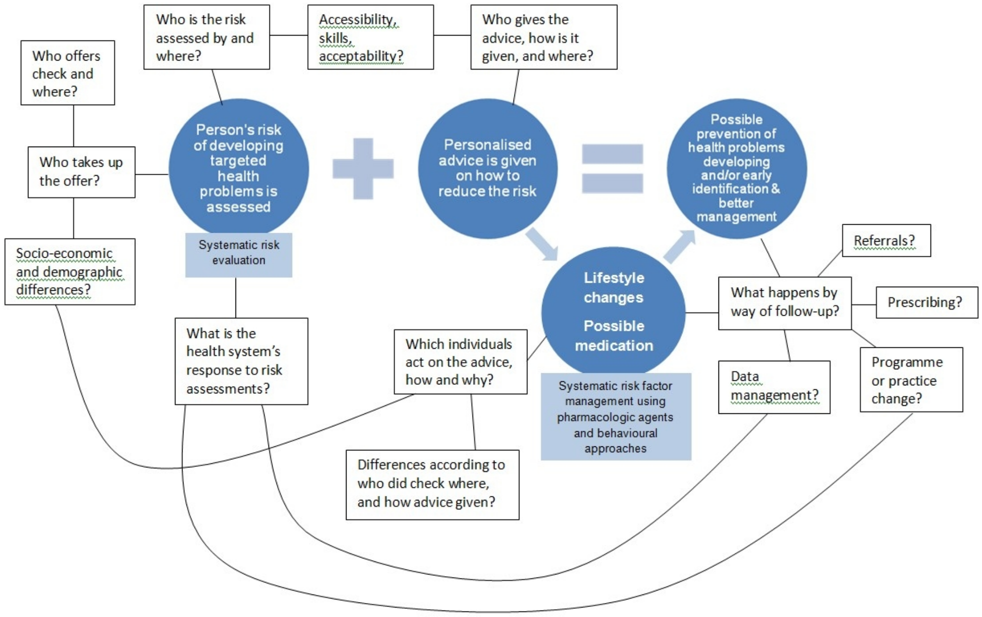

Figure 2: Initial programme theory behind NHS Health Checks and some of the processes that may influence outcomes

296x188mm (96 x 96 DPI)

Supplementary File

Search strategies

The full details of the search strategies for **Step 2a** of our protocol paper is provided below.

MEDLINE (via Ovid)

1	health check*.ti,ab,kw
2	(NHS OR National Health Service OR United Kingdom OR UK OR England OR English).ti,ab,kw
3	exp England/
4	2 or 3
5	1 and 4
6	limit 5 to (english language and yr="2008-Current")

Embase (via Ovid)

1	health check*.ti,ab,kw
2	(NHS OR National Health Service OR United Kingdom OR UK OR England OR English).ti,ab,kw
3	england/
4	2 or 3
5	1 and 4
6	limit 5 to (english language and yr="2008-Current")

CINAHL (via EbscoHost)

S1	TX "health check*"
S2	TI (NHS OR "National Health Service" OR "United Kingdom" OR UK OR England OR English) OR AB (NHS OR "National Health Service" OR "United Kingdom" OR UK OR England OR English)
S3	MH "England"
S4	S2 OR S3
S5	S1 AND S4
S6	Limiters: Published Date: 20080101-20201231; English Language; Expanders: Apply equivalent subjects

HMIC (via Ovid)

1	health check*.mp
2	(NHS OR National Health Service OR United Kingdom OR UK OR England OR English).mp
3	exp england/
4	exp health authorities in england/
5	or/2-4
6	1 and 5
7	limit 6 to (yr="2008-Current")

Web of Science Citation Indexes (SCI-EXPANDED, SSCI)

#1	TOPIC: ("health check*")

For peer review only

#2	TOPIC: (NHS OR "National Health Service" OR "United Kingdom" OR UK OR England OR English)
#3	#1 and #2
#4	Refined by: PUBLICATION YEARS: (2020 OR 2012 OR 2019 OR 2011 OR 2018 OR 2010 OR 2017 OR 2009 OR 2016 OR 2008 OR 2015 OR 2014 OR 2013) AND LANGUAGES: (ENGLISH)

For peer review only

Reporting checklist for protocol of a systematic review.

Based on the PRISMA-P guidelines.

Instructions to authors

Complete this checklist by entering the page numbers from your manuscript where readers will find each of the items listed below.

Your article may not currently address all the items on the checklist. Please modify your text to include the missing information. If you are certain that an item does not apply, please write "n/a" and provide a short explanation.

Upload your completed checklist as an extra file when you submit to a journal.

In your methods section, say that you used the PRISMA-Preporting guidelines, and cite them as:

Moher D, Shamseer L, Clarke M, Ghersi D, Liberati A, Petticrew M, Shekelle P, Stewart LA. Preferred Reporting Items for Systematic Review and Meta-Analysis Protocols (PRISMA-P) 2015 statement. Syst Rev. 2015;4(1):1.

		Reporting Item	Page Number
**Title**			
Identification	#1a	Identify the report as a protocol of a systematic review	1
Update	#1b	If the protocol is for an update of a previous systematic review, identify as such	n/a
**Registration**			
	#2	If registered, provide the name of the registry (such as PROSPERO) and registration number	2
**Authors**			
Contact	#3a	Provide name, institutional affiliation, e-mail address of all protocol authors; provide physical mailing address of corresponding author	1
Contribution	#3b	Describe contributions of protocol authors and identify the guarantor of the review	16
**Amendments**			

For peer review only

	#4	If the protocol represents an amendment of a previously completed or published protocol, identify as such and list changes; otherwise, state plan for documenting important protocol amendments	n/a
**Support**			
Sources	#5a	Indicate sources of financial or other support for the review	16
Sponsor	#5b	Provide name for the review funder and / or sponsor	16
Role of sponsor or funder	#5c	Describe roles of funder(s), sponsor(s), and / or institution(s), if any, in developing the protocol	n/a
**Introduction**			
Rationale	#6	Describe the rationale for the review in the context of what is already known	3-7
Objectives	#7	Provide an explicit statement of the question(s) the review will address with reference to participants, interventions, comparators, and outcomes (PICO)	8
**Methods**			
Eligibility criteria	#8	Specify the study characteristics (such as PICO, study design, setting, time frame) and report characteristics (such as years considered, language, publication status) to be used as criteria for eligibility for the review	12
Information sources	#9	Describe all intended information sources (such as electronic databases, contact with study authors, trial registers or other grey literature sources) with planned dates of coverage	9-10
Search strategy	#10	Present draft of search strategy to be used for at least one electronic database, including planned limits, such that it could be repeated	n/a - not yet developed
Study records - data management	#11a	Describe the mechanism(s) that will be used to manage records and data throughout the review	12-13
Study records - selection process	#11b	State the process that will be used for selecting studies (such as two independent reviewers) through each	12

For peer review only

		phase of the review (that is, screening, eligibility and inclusion in meta-analysis)	
Study records - data collection process	#11c	Describe planned method of extracting data from reports (such as piloting forms, done independently, in duplicate), any processes for obtaining and confirming data from investigators	13
Data items	#12	List and define all variables for which data will be sought (such as PICO items, funding sources), any pre-planned data assumptions and simplifications	13
Outcomes and prioritization	#13	List and define all outcomes for which data will be sought, including prioritization of main and additional outcomes, with rationale	13
Risk of bias in individual studies	#14	Describe anticipated methods for assessing risk of bias of individual studies, including whether this will be done at the outcome or study level, or both; state how this information will be used in data synthesis	n/a. Relevance and rigour will be assessed as is appropriate for realist review (see page 13)
Data synthesis	#15a	Describe criteria under which study data will be quantitatively synthesised	n/a
Data synthesis	#15b	If data are appropriate for quantitative synthesis, describe planned summary measures, methods of handling data and methods of combining data from studies, including any planned exploration of consistency (such as I2, Kendall's τ)	n/a
Data synthesis	#15c	Describe any proposed additional analyses (such as sensitivity or subgroup analyses, meta-regression)	n/a
Data synthesis	#15d	If quantitative synthesis is not appropriate, describe the type of summary planned	13-14
Meta-bias(es)	#16	Specify any planned assessment of meta-bias(es) (such as publication bias across studies, selective reporting within studies)	n/a
Confidence in cumulative evidence	#17	Describe how the strength of the body of evidence will be assessed (such as GRADE)	n/a

Notes:

For peer review only

- 10: n/a - not yet developed
- 14: n/a. Relevance and rigour will be assessed as is appropriate for realist review (see page 13) The PRISMA-P checklist is distributed under the terms of the Creative Commons Attribution License CC-BY 4.0. This checklist was completed on 11. January 2021 using https://www.goodreports.org/, a tool made by the EQUATOR Network in collaboration with Penelope.ai

For peer review only
