# Supplementary material for: NHS Health Check programme: a protocol for a realist review
Source: BMJ Open. 2021 Apr 14;11(4):e048937. doi: 10.1136/bmjopen-2021-048937 (PMC8054100; doi:10.1136/bmjopen-2021-048937)
Supplement: Reviewer comments [file bmjopen-2021-048937.reviewer_comments.pdf]

PEER REVIEW HISTORY

BMJ Open publishes all reviews undertaken for accepted manuscripts. Reviewers are asked to complete a checklist review form (**http://bmjopen.bmj.com/site/about/resources/checklist.pdf**) and are provided with free text boxes to elaborate on their assessment. These free text comments are reproduced below.

ARTICLE DETAILS

**TITLE (PROVISIONAL)**	NHS Health Check Programme: protocol for a realist review
**AUTHORS**	Duddy, Claire; Wong, Geoff; Gadsby, E; Krska, Janet; Hibberd, Vivienne

This article was not externally reviewed at BMJ Open. Protocols that have been independently assessed prior to submission to BMJ Open are usually fast-tracked to publication on the grounds that further substantial changes will not be possible. This independent assessment will usually be external, independent review for both a substantial grant award from a non-commercial or government funder and ethics approval.
